# “Are More Cues Always Better?” Effects of Cue-Based Instructional Support on Chinese L2 Vocabulary Processing and Immediate Learning Outcomes: Eye-Tracking Evidence

**DOI:** 10.3390/bs16060962

**Published:** 2026-06-10

**Authors:** Yu Yuan, Jinqiao Zhang, Yunxiao Ma, Lixuan Huang

**Affiliations:** 1 Department of Applied Linguistics, College of Chinese Language and Culture, Jinan University, Guangzhou 510610, China; 2 Linguistics and Sociology, School of Arts, Languages and Cultures, Faculty of Humanities, The University of Manchester, Oxford Road, Manchester M13 9PL, UK

**Keywords:** cue-based instructional support, L2 Chinese vocabulary learning, linguistic–semantic support, typographical enhancement, eye tracking

## Abstract

Grounded in the Cognitive Theory of Multimedia Learning and Cognitive Load Theory, this study examined how cue-based instructional support relates to L2 Chinese vocabulary processing and immediate learning outcomes. Forty intermediate-to-advanced learners studied 24 disyllabic pseudowords under four within-subject conditions: no cueing, verbal cueing (linguistic–semantic support via definitions and collocations), physical cueing (typographical enhancement via bolded targets and underlined contextual words), and full cueing. Eye movements, immediate post-tests, and questionnaires were analyzed. The results revealed selective, measure-dependent effects rather than uniform facilitation. In the Orthographic Choice Task, no cueing outperformed full cueing. In the Semantic Priming Decision Task, verbal cueing yielded a higher accuracy than physical cueing, indicating that linguistic–semantic support benefited initial meaning-related processing more than typographical enhancement. No differences emerged in the Sentence Acceptability Judgment Task. Eye-tracking showed shorter first fixations under physical than verbal cueing, suggesting the limited facilitation of early visual orienting. Full cueing showed no consistent advantage over verbal cueing but elicited larger pupil sizes and longer total fixation durations on targets, indicating additional coordination demands. Learners most often preferred full-cueing materials, yet rated verbal cueing as most helpful. An effective cue-based design should align the cue format and content with the target learning dimension while avoiding unnecessary processing demands. The findings reflect immediate learning under controlled conditions rather than long-term acquisition.

## 1. Introduction

Vocabulary is the foundation of language mastery and a central concern in Chinese language learning (Schmitt, 2000). In second language (L2) vocabulary acquisition research, vocabulary learning is commonly divided into intentional and incidental learning (Hulstijn, 2001; Nation, 2022). Intentional vocabulary learning refers to the process by which learners directly acquire the form—both phonological and orthographic—and the meaning of lexical items through deliberate, explicit activities such as memorizing word lists or flashcards, consulting dictionaries, and completing vocabulary exercises (Hunt & Beglar, 2005). This approach emphasizes learners’ conscious attention to lexical form and meaning and fosters acquisition through systematic activities involving repeated exposure and recycling, thereby supporting long-term retention (Hulstijn, 2001; Schmitt, 2008). From a pedagogical standpoint, this explicit focus on linguistic form can be regarded as a manifestation of form-focused instruction (Ellis, 2001) and represents an important component of L2 vocabulary classroom instruction, enabling learners to accumulate high-frequency words relatively rapidly and laying the groundwork for the subsequent development of reading comprehension (Hunt & Beglar, 2005; Laufer, 1997).

### 1.1. Cueing Effects in Vocabulary Learning

With the advancement of information technology, multimedia has been increasingly applied to second language vocabulary instruction. In multimedia learning, “cues” refer to instructional elements that enhance learning outcomes by guiding attention and thought, highlighting key structures, easing comprehension, helping learners select critical information, and facilitating the encoding, retrieval, organization, and integration of information (Fiorella & Mayer, 2015). When learners draw on such cues to support target vocabulary learning, the resulting facilitation is termed the cueing effect. Recent work on technology-enhanced vocabulary learning has emphasized the need to move beyond the general question of whether technology or multimedia support is effective and to examine how specific design features support different dimensions of vocabulary knowledge. For example, Zhou et al. (2024) found that much of the existing research in this area has focused primarily on receptive knowledge and vocabulary breadth, underscoring the need for more balanced attention to vocabulary depth, productive knowledge, and theory-driven instructional design. This observation is particularly relevant to the present study, which examines cue-based support across the orthographic, semantic, and usage-related dimensions of Chinese L2 vocabulary learning.

Some researchers have argued that cues exist in multiple forms—such as spoken language, text, or images—and that they help learners allocate attentional resources, thereby reducing interference in the learning environment (Mayer, 2021). Empirical studies have shown that learning materials containing cues promote learning outcomes more effectively than those without (Albus et al., 2021; de Koning et al., 2007; Hu & Zhang, 2021; Huang et al., 2024; Jamet et al., 2008). Meta-analytic findings further indicate that cues enhance retention and transfer to some extent by reducing cognitive load during learning (Xie et al., 2017).

The Cognitive Theory of Multimedia Learning (CTML; Mayer, 2021) posits that learners process multimedia information through three successive stages: selecting, organizing, and integrating. In the context of direct vocabulary learning, this processing chain can be specified as follows: learners first select form- and meaning-relevant information about target words from multimodal materials, then organize it into a coherent internal structure in working memory, and, finally, integrate the new information with their existing linguistic knowledge, ultimately forming more stable lexical representations. Cues are thought to enhance learning by exerting a sustained influence across all three stages.

During the selecting stage, cues direct learners’ attention toward locations and information critical for comprehension and mental representation more effectively than non-cued materials do (de Koning et al., 2009; Hu & Zhang, 2021; Jamet et al., 2008; Mautone & Mayer, 2001). Eye-tracking research has further shown that the coordination of visual and auditory cues can significantly enhance attentional allocation to key information (Xie et al., 2019), thereby optimizing information selection. During the organizing stage, visual and combined audiovisual cues have been found to guide attention and reduce the cognitive load (Huang et al., 2024), while formats such as eye-movement modeling examples promote the structured processing of information (F. Wang et al., 2020). During the integrating stage, visual cues have been shown to significantly facilitate learners’ deep processing of the material (Albus et al., 2021).

Other studies, however, indicate that the presence of cues does not necessarily improve performance and may even increase the cognitive burden. Some have found that cued materials neither reduced the subjectively reported cognitive load nor lowered the perceived task difficulty (de Koning et al., 2010; Richter et al., 2016). Cued materials have also failed to reliably improve learning outcomes in a number of studies (Harp & Mayer, 1998; Lin & Atkinson, 2011; Ozcelik et al., 2010; Rey, 2010), and arrow cues, in particular, have proven ineffective in guiding attention or facilitating learning (Boucheix & Lowe, 2010; Kriz & Hegarty, 2007).

The Limited Capacity Hypothesis (Kahneman, 1973; Navon & Gopher, 1979) and Cognitive Load Theory (CLT; Sweller, 1988; Sweller et al., 1998) hold that human cognitive resources are finite and that processing efficiency declines when task demands exceed available resources. van Gog (2014) noted that, when verbal and visual cues are presented simultaneously in a novel learning environment, learners may struggle to integrate the two, with adverse effects on learning.

More recent research has extended this line of reasoning. In a study of instructional animations, Arslan-Ari et al. (2020) found that different cueing conditions produced no significant effects on learning outcomes or mental effort, and that high-prior-knowledge learners in the no-cueing condition actually achieved higher transfer and matching scores. Moon and Ryu (2021) reported that social cues can cause visual distraction, impair comprehension, and increase cognitive load. Other research has shown that different types of cues vary in effectiveness and may even produce interference under certain conditions (Li et al., 2023), further indicating that cueing effects are markedly context-dependent.

A review of this literature reveals a notable gap. The existing cueing research has focused primarily on facilitative effects in general knowledge learning, with comparatively little attention to the differential roles and potential interactions of distinct cue types in second language vocabulary learning (Jamet et al., 2008; Lin & Atkinson, 2011). Moreover, because previous studies have operationalized verbal, visual, and physical cues in different ways, it is necessary to clarify how these conditions are defined in the present study before examining their differential effects.

### 1.2. Operationalizing Verbal and Physical Cueing in L2 Vocabulary Learning

Although a unified classification system for cues has yet to be established, multimedia learning researchers typically distinguish between verbal signaling and visual signaling, which correspond to two distinct but complementary processing pathways—meaning support and attention guidance, respectively (Beege et al., 2024; de Koning et al., 2009; Mautone & Mayer, 2001).

In what follows, we use the term “cueing” rather than “signaling” because the verbal support examined in this study supplies substantive lexical–semantic content and thus extends beyond the attention-highlighting function typically denoted by “signaling”. Because cueing has been used differently across multimedia learning and vocabulary learning studies, we first clarify how it is conceptualized here. In the vocabulary learning context addressed in this study, cueing is broadly defined as instructional support that guides learners’ attention to task-relevant lexical information. Within this framework, we operationalize verbal support as comprising two types of instructional content: (1) definitions, which facilitate form–meaning mapping by providing explicit explanations of word meanings (Nation, 2022); and (2) collocations, which reveal usage conditions and typical semantic relationships (Boers & Lindstromberg, 2009; Webb, 2007).

We adopt the term “verbal cueing” to maintain consistency with the four experimental conditions, while explicitly acknowledging that it departs from its traditional usage in multimedia learning research. Strictly speaking, definitions and collocations constitute substantive instructional input that supplies semantic and usage knowledge, rather than attentional cues in the traditional sense—that is, elements that highlight or direct attention to existing information without adding new content. This distinction matters: verbal support in the present study operates by providing substantive lexical content, not merely by signaling where to attend, and this content-provision mechanism differs fundamentally from the perceptual-salience mechanism characteristic of physical cueing. The interpretation is also consistent with recent glossing research showing that the level and richness of gloss information can shape vocabulary learning outcomes. Rassaei and Folse (2024), for instance, found that sentence-level L2 glosses were more effective than L1 or L2 word-level glosses, suggesting that contextualized linguistic support may foster richer form–meaning connections than more limited lexical support. In the present study, definitions and collocations likewise provided learners with explicit semantic and usage-related information and therefore functioned as lexical–semantic instructional support rather than as pure attentional cues.

Although the present study adopts this operationalization, the broader literature on verbal cueing remains far from settled, and many questions are still unresolved (Boers, 2022). First, its conceptual definition remains blurred: studies vary in their understanding of the construct, often conflating structural guidance, explanatory text, personalized expressions, and even social prompts, which undermines the comparability across findings (Mayer, 2021). Second, verbal cues often appear as additional textual information, and, if their length, density, and position are not strictly controlled, differences in the information load may arise, making it difficult to attribute experimental effects solely to the cues themselves (Zhang et al., 2025; van Gog, 2021). Third, a gap persists between experimental settings and authentic classroom instruction, so the ecological validity of these findings requires further verification (Tabbers et al., 2004). The independent role and boundary conditions of verbal cueing in L2 vocabulary learning therefore remain to be clarified.

Visual signaling (e.g., bolding, underlining, highlighting, and color) can effectively focus learners’ attentional resources on key areas, supporting the selection, organization, and integration of relevant information and improving retention and transfer under certain conditions (de Koning et al., 2009; Jamet et al., 2008; Lin & Atkinson, 2011). It is therefore one of the more extensively researched cue types. Some studies, however, indicate that, although visual cues can enhance the attention to and processing of target items, their effect on learning outcomes is typically small to medium in magnitude, and combining multiple enhancement formats does not necessarily outperform a single format (Lee & Huang, 2008).

Several issues nonetheless remain unresolved. First, in processing textual materials, previous studies have frequently confounded visual and verbal cues, producing a substantial variation in the effects depending on the operational methods and research designs (Han et al., 2008). Second, the research on vocabulary acquisition has paid insufficient attention to cues such as bolding and underlining, often subsuming them under the broad category of salience enhancement. In vocabulary learning, however, these cues serve two specific functions: they increase the visual contrast between the target word and the surrounding text, allowing it to stand out and thereby prioritizing the learner’s attention (Lorch, 1989); and they help learners extract key information and ignore interference when multiple competing cues are present (Cintrón-Valentín & Ellis, 2016).

Building on this discussion, physical cueing in the present study refers specifically to typographical enhancement, such as bolding target words and underlining relevant contextual words. The term emphasizes that such cues function primarily through the physical presentation characteristics of the target item rather than through additional linguistic content.

In previous studies, verbal and physical cues have often been embedded together as composite forms, making it difficult to distinguish their independent roles and relative contributions (Richter et al., 2016). As Zou and Teng (2023) noted in their work on multimedia-assisted vocabulary learning, multidimensional support is often presented in an intertwined manner, which makes it challenging to isolate how a single cue contributes to vocabulary gains; in the absence of the systematic separation of each component, the underlying mechanisms remain unclear (Alpizar et al., 2020).

According to multimedia cueing theories and attention-guidance frameworks, visual cues act more directly on the selection stage, whereas verbal cues are more likely to support organization and integration; the two therefore have theoretical potential to complement each other (de Koning et al., 2009; van Gog, 2021). Such complementarity, however, does not entail that simultaneous presentation is necessarily superior to a single cue. In their study of L2 grammar and vocabulary learning, Cintrón-Valentín and García-Amaya (2021) found that combining physical and verbal cues can provide stronger formal salience in multimodal learning, but that this effect is not unconditionally stable, depending instead on the target items, task demands, and measurement indices. The experimental and meta-analytic evidence likewise suggests that using multiple cues simultaneously does not always confer additional benefits and may even weaken learning through cue redundancy or increased processing burden (Xie et al., 2019). The effectiveness of combined cueing may vary with the learning task, presentation mode, and measurement dimension (Jung et al., 2025b; Majuddin et al., 2021; Montero Perez et al., 2015; Révész et al., 2023). In vocabulary learning specifically, if verbal cues supply extensive explanatory information while physical cues continuously reinforce explicit attention, learners may direct their limited cognitive resources toward maintaining surface-level attention rather than coordinating these inputs to establish deep connections among word form, meaning, and usage.

In summary, although the existing research shows that cues can promote learning, their effects are unstable and vary with cue type, task context, informational complexity, and measurement timing. With respect to L2 vocabulary learning, three gaps remain. First, few studies have systematically compared no cueing, single physical cueing, single verbal cueing, and their combination within a single framework, making it difficult to distinguish the relative roles of attention guidance and semantic support. Second, vocabulary learning outcomes are often treated as a unitary construct, with little investigation of the differences across dimensions such as meaning comprehension and collocation mastery. Third, learners’ subjective experiences have received insufficient attention. Multimedia learning research has shown that cues affect not only the learning performance but also processing experiences such as mental effort, and examining these subjective measures alongside objective outcomes would yield a more comprehensive understanding of the mechanisms underlying cueing effects (Crooks et al., 2012). Building on these considerations, a systematic comparison of different cueing conditions in L2 intentional vocabulary learning is needed to clarify the functional characteristics of each cue type and their potential combined benefits.

### 1.3. The Present Study

To address these gaps, the present study examined how different forms of cue-based instructional support are associated with L2 Chinese vocabulary processing and immediate learning outcomes. Rather than treating cueing as a unitary construct, we distinguish between lexical–semantic support and typographical enhancement. Lexical–semantic support (verbal cueing) provides additional lexical information through meaning-related explanations and collocations, whereas typographical (physical cueing) enhancement increases the perceptual salience of target items through devices such as bolding and underlining.

This distinction is particularly relevant for L2 Chinese, because Chinese characters differ substantially from alphabetic scripts in their orthographic structure. Whereas alphabetic word learning typically relies on relatively systematic grapheme–phoneme mappings, character learning requires attention to visually complex stroke configurations, and radicals, and spatial arrangements, as well as to mappings among written form, syllable or morpheme, meaning, and contextual use. For L2 learners from non-character-based L1 backgrounds, this orthographic dissimilarity may heighten the demands of encoding visual-form information while simultaneously establishing semantic and usage representations. Research on Chinese as a second language has similarly shown that character- and word-level features are an important source of difficulty for learners from diverse L1 backgrounds, especially when their first-language writing systems are orthographically distant from Chinese (Yan & Lin, 2023). Moreover, recent eye-tracking evidence indicates that multimodal annotation can facilitate Chinese L2 vocabulary acquisition and alter online visual processing patterns (Y. Wang & Xu, 2025). These findings reinforce the need to examine how different forms of instructional support guide learners’ processing of Chinese vocabulary.

Within this context, the two types of support are likely to operate at different processing stages. Typographical enhancement, such as bolding and underlining, may function primarily at the selection stage, directing early visual attention to target forms and informative contextual cues. Lexical–semantic support, such as definitions and collocations, may contribute more directly to organization and integration by making the form–meaning and form–use mappings explicit. The orthographic specificity of Chinese further suggests that combined cueing may not yield additive benefits, since learners must coordinate visually complex orthographic input with semantic and usage information under a limited working-memory capacity.

To examine these possibilities, the study combined eye-tracking measures with immediate post-test performance, thereby capturing both online processing and short-term learning outcomes under controlled experimental conditions. The eye-tracking measures the indexed learners’ allocation of visual attention and processing effort during vocabulary exposure, whereas the behavioral tasks assessed the immediate recognition, semantic processing, and sentence-level use of the target items. Because the two forms of support differ in both perceptual form and informational content, condition differences are interpreted as reflecting instructional-support functions rather than cue format alone.

### 1.4. Research Hypotheses

Drawing on CTML, CLT, and prior research on cueing and vocabulary learning, we proposed the following hypotheses. They concern how behavioral performance, eye-tracking patterns, and learner evaluations jointly reflect the effects of different forms of cue-based instructional support. The no-cueing condition served as the baseline for evaluating the added value of verbal, physical, and combined cueing.

**H1.**  *Verbal cueing as lexical–semantic support.*

If verbal cueing yields better immediate vocabulary outcomes than no cueing, a stronger semantic and usage-related performance than physical cueing, and a longer dwell time and more fixations on the learning materials, this pattern would support the CTML-based view that lexical–semantic support promotes deeper form–meaning mapping and elaborative processing. More favorable learner evaluations would further indicate that definitions and collocations are perceived as useful vocabulary-learning support.

Conversely, if verbal cueing increases the dwell time, fixation counts, or pupil size without improving behavioral performance or learner evaluations, this pattern would suggest that the added semantic and collocational information increases the cognitive load rather than facilitating learning.

**H2.**  *Physical cueing as typographical/perceptual support.*

If physical cueing improves form-related performance relative to no cueing and facilitates early visual processing—as reflected in the shorter first-fixation duration, shorter first-run dwell time on target words, or more efficient attention to visually marked areas—this pattern would support the signaling principle. Positive learner evaluations would further suggest that typographical enhancement is perceived as a useful attentional aid.

Conversely, if physical cueing increases the overall dwell time, fixation counts, or pupil size without corresponding behavioral or evaluative benefits, this pattern would suggest that visual enhancement alone may impose additional perceptual demands or offer limited instructional value.

**H3.**  *Full cueing as combined lexical–semantic and typographical support.*

If full cueing produces the strongest immediate vocabulary outcomes, receives the most favorable learner evaluations, and shows coordinated attention to target words, semantic information, and highlighted contextual cues, this pattern would support the CTML-based view that lexical–semantic and perceptual support can jointly facilitate vocabulary processing.

If, however, full cueing does not outperform verbal cueing alone, or if it elicits a greater dwell time, more fixations, and a larger pupil size without additional behavioral or evaluative benefits, this pattern would support a CLT-based interpretation: combined cues may increase coordination demands or cognitive-resource competition rather than produce additive benefits.

## 2. Method

### 2.1. Participants

Forty L2 Chinese learners enrolled at the College of Chinese Language and Culture, Jinan University, participated in the experiment. One participant was excluded from the final analyses because of poor eye-tracking data quality, including a high proportion of invalid samples. Therefore, the final analyses of behavioral data, eye-tracking data, and questionnaire data were based on 39 participants.

The final sample included 14 male and 25 female participants. Their mean age was 22.4 years (*SD* = 2.4), with an age range of 19 to 31 years. Participants came from multiple countries, including Indonesia (*n* = 21), Vietnam (*n* = 5), Myanmar (*n* = 3), Thailand (*n* = 3), Peru (*n* = 2), Panama (*n* = 1), the Philippines (*n* = 1), Kyrgyzstan (*n* = 1), Laos (*n* = 1), and Mauritius (*n* = 1). All participants were right-handed and had normal or corrected-to-normal vision. Participants also reported their first-language backgrounds. The final sample included nine L1 backgrounds: Indonesian, Vietnamese, Thai, Burmese, Lao, Spanish, French, Uzbek, and Filipino. These L1s differed in both language family and writing system. Indonesian, Vietnamese, Spanish, French, Uzbek, and Filipino are primarily written with alphabetic scripts, whereas Thai, Burmese, and Lao use Brahmic-derived alphasyllabic writing systems. None of the participants reported an L1 writing system that is morphosyllabic or logographic in the same sense as Chinese characters. Thus, although the sample was linguistically diverse, all participants encountered Chinese characters as an orthographically distant writing system relative to their L1 literacy background, albeit to varying degrees.

All participants had obtained a certificate of the Hanyu Shuiping Kaoshi (HSK; Chinese Proficiency Test) at Level 5 or above, or had obtained an HSK Level 4 certificate one to two years prior to the study. Under the three-band, nine-level framework of the Chinese Proficiency Grading Standards, HSK Levels 1–3 correspond to elementary proficiency, Levels 4–6 to intermediate proficiency, and Levels 7–9 to advanced proficiency. Participants were therefore considered intermediate-to-advanced L2 Chinese learners. Their Chinese-learning experience ranged from 2 to 17 years, including formal classroom instruction, family-based learning, and after-school tutoring. Although participants varied in their learning histories, their current proficiency was further assessed using a cloze test.

A cloze test developed by Feng et al. (2020) was directly adopted as the pre-test instrument (see the Appendix A for the complete list). The test was constructed using the fixed-ratio deletion method, in which every nth word was systematically deleted from a 301-character passage to generate 30 blanks. All participants completed the test, with a full score of 30 points. The mean score was 25.6 (*SD* = 2.7), the mode was 27, and scores ranged from 20 to 30. These results further indicated that the participants had relatively comparable intermediate-to-advanced Chinese proficiency.

An a priori power analysis was conducted using G*Power 3.1 (Faul et al., 2009) before data collection to determine the required sample size. The analysis was based on a repeated-measures ANOVA with one within-subjects factor and four levels. Assuming a medium effect size of *f* = 0.25, an alpha level of 0.05, desired power of 0.80, one group, four repeated measurements, a mean correlation of 0.50 among repeated measures, and a nonsphericity correction of *ε* = 1, the analysis indicated that a minimum sample size of 24 participants was required. To allow for possible data loss in eye-tracking recording, 40 participants were recruited. One participant was excluded from the final analyses due to substantial data loss and poor eye-tracking quality, yielding a final sample of 39 participants. The final analytical sample of 39 participants still exceeded the required minimum sample size for detecting medium-sized within-subjects effects.

### 2.2. Experimental Design and Instructional-Support Conditions

Drawing on the foregoing literature review and the operational definition of cueing adopted in this study, the experiment focuses on two forms of cue-based instructional support: verbal cueing and physical cueing. Verbal cueing was operationalized as linguistic–semantic support whereas physical cueing was operationalized as perceptual cueing through typographical enhancement. This distinction allowed the study to examine the relative contributions of linguistic–semantic support and perceptual attention guidance in Chinese L2 vocabulary learning, as well as whether their concurrent presentation produces additional benefits or increased coordination demands. To minimize interference from extraneous modality factors, no other forms of support such as audio or animation are introduced.

On this basis, four instructional-support conditions were established: no cueing (baseline), verbal cueing (linguistic–semantic cueing), physical cueing (perceptual cueing), and full cueing (combined cueing). In the no cueing condition, target words were presented in sentence contexts without additional lexical–semantic information or typographical enhancement. In the verbal cueing condition, target words were accompanied by linguistic–semantic support. In the physical cueing condition, target words were visually enhanced through typographical marking. In the full cueing condition, linguistic–semantic support and typographical enhancement were presented concurrently.

An important feature of this design is that the four conditions form two information-load levels. The no cueing and physical cueing conditions provided limited lexical information, whereas the verbal cueing and full cueing conditions provided richer lexical–semantic information. This structure has important interpretive implications. Comparisons between the no cueing and physical cueing conditions allow the contribution of typographical enhancement to be examined while holding verbal information constant. Similarly, comparisons between the verbal cueing and full cueing conditions examine whether adding physical cueing to information-rich verbal support produces additional benefits or increased processing demands. By contrast, comparisons across information-load levels should be interpreted as comparisons between realistic instructional-support options that differ in both cue format and lexical–semantic information. Thus, the present design compares different forms of cue-based instructional support while acknowledging that cueing format and informational richness are not fully orthogonal across all conditions.

### 2.3. Materials and Presentation Format

#### 2.3.1. Learning Phase

The learning materials consisted of target pseudowords, example sentences, L2 definitions, and collocations. The selection of these elements was informed by the view that vocabulary knowledge involves multiple dimensions, including form, meaning, and use (Nation, 2022), and that L2 vocabulary learning requires learners to establish form–meaning mappings and develop contextualized knowledge of word use (Richards, 1976; Webb, 2007). Example sentences served as the foundational learning material across all experimental conditions, providing contextual information for target-word processing. L2 definitions and collocations constituted the verbal cues by supplying semantic explanations and usage-related information, whereas bolding and underlining served as physical cueing by increasing the perceptual salience of the target pseudowords and relevant contextual information.

**Target words.** Twenty-four disyllabic words were selected as target words from the Chinese Proficiency Grading Standards for International Chinese Language Education (Center for Language Education and Cooperation, 2021). All target words were low-frequency advanced-level words (Levels 7–9), comprising eight verbs, eight nouns, and eight adjectives. In the formal experiment, the target words were replaced by disyllabic pseudowords constructed from rare characters; for example, “颁布 (/ban1bu4/, promulgate)” was replaced by “帠卪 (/yi4jie2/)”. Two Chinese language teachers with more than five years of teaching experience selected rare characters from the Table of General Standard Chinese Characters (State Council of the People’s Republic of China, 2013). Characters with radicals or components commonly encountered in instruction or daily life were excluded. The orthographic structure and stroke count of the pseudowords were controlled to ensure no significant differences across conditions; the mean stroke count per character was 11.1 (*SD* = 0.3).

**Example sentences.** Example sentences served as the experimental baseline and were compiled with reference to the Contemporary Chinese Dictionary (7th ed.; Institute of Linguistics, Chinese Academy of Social Sciences Dictionary Editorial Office, 2016), the Commercial Press Learner’s Dictionary of Chinese (Commercial Press Dictionary Research Center, 2006), and the BCC Corpus (Xun et al., 2016). All example sentences were compound sentences in which the character counts of the preceding and following clauses were equal, totaling 14 characters per sentence. Sentences sharing the same part-of-speech category had identical grammatical structures, with the target word appearing in the same syntactic position. Upon completion, five Chinese language teachers with more than five years of teaching experience were invited to evaluate and review the sentences. Additionally, 10 native speakers and 10 L2 learners who did not participate in the experiment rated the match between each definition and its corresponding target word, as well as the familiarity of each collocation. The mean rating for definition-target word match was 8.38 (*SD* = 0.55), and the mean collocation familiarity rating was 8.79 (*SD* = 0.44).

To control for the properties of the experimental materials, the contextual contribution of all example sentences was assessed to ensure a consistent level of contextual richness. The calculation method followed Y. Wang and Xu (2023): native speakers were invited to rate the degree to which each sentence’s context facilitated inference of the target word on a 0–10 scale, and a sentence’s contextual contribution score was defined as the mean of all ratings. After repeated revision and evaluation, the contextual contribution scores of all sentences fell within the range of 6.5–7.5, with a mean of 7.01, indicating a contextual contribution rate of 70%. Subsequently, the text difficulty of all experimental sentences was analyzed using the text readability grading system developed by Cheng et al. (2023) based on the Chinese Proficiency Grading Standards for International Chinese Language Education. This system automatically evaluates and outputs a difficulty score and a corresponding proficiency level within a “three-band, nine-level” framework by analyzing the grade distribution of characters, vocabulary, and grammatical points in the text. The analysis revealed that the mean text difficulty score for all experimental sentences was 2.0 (*SD* = 0.29), placing them within the elementary range.

**Verbal cueing.** Verbal cueing comprised definitions and collocations. Definitions were drawn from the Contemporary Chinese Dictionary (7th ed.) and the Commercial Press Learner’s Dictionary of Chinese, and were subsequently adapted to a length of 7–9 characters. Collocations were sourced from the Contemporary Chinese Dictionary (7th ed.), the Commercial Press Learner’s Dictionary of Chinese, and the BCC Corpus, and were fixed at four characters in length. Following compilation, the materials were evaluated and revised by five Chinese language teachers with teaching experience, and subsequently rated by 10 international students and 10 native speakers who did not participate in the experiment for match quality and familiarity. The mean definition match rating was 8.38 (*SD* = 0.55), and the mean collocation familiarity rating was 8.79 (*SD* = 0.44).

**Physical cueing.** Physical cueing consisted of bolding and underlining. Target pseudowords in the example sentences were bolded, and contextually informative words or phrases that could help learners infer the meaning or usage of the target pseudowords were underlined. These underlined words or phrases were treated as part of the physical cueing manipulation because they visually highlighted key contextual information relevant to target-word comprehension. The final underlining positions were determined through evaluation by 10 native speakers. Target pseudowords appeared in the middle of the experimental sentences (Zang et al., 2020). For example, “国家**帠卪**新计划，吸引海内外人才。” (The country [帠卪, means promulgate] a new plan to attract talent from home and abroad.), the target pseudoword “帠卪” replaced the original word “颁布” and was bolded, while “吸引” [means attract] was underlined because it provided key contextual information for interpreting the sentence. Both forms of visual marking were implemented in the physical cueing conditions.

**Attention check items.** To ensure that participants engaged attentively throughout the experiment, multiple-choice questions appeared after every six target word learning trials, requiring participants to identify a word they had just studied.

**Material presentation.** Four types of materials were presented, corresponding to the four instructional-support conditions: Condition 1 (no cueing), Condition 2 (verbal cueing), Condition 3 (physical cueing), and Condition 4 (full cueing). All materials followed a uniform layout: the target pseudoword appeared before a colon, and the three lines following the colon corresponded, respectively, to the definition, the collocations, and the example-sentence positions.

Conditions 1 and 3 presented only the target pseudoword and an example sentence, with placeholder symbols (“X”) occupying the definition and collocation positions. Conditions 2 and 4 presented additional linguistic–semantic information, with the definition and two collocations displayed in the corresponding positions. In Conditions 3 and 4, physical cueing was implemented by bolding the target pseudoword and underlining informative contextual words to enhance their perceptual salience.

**Figure 1:**
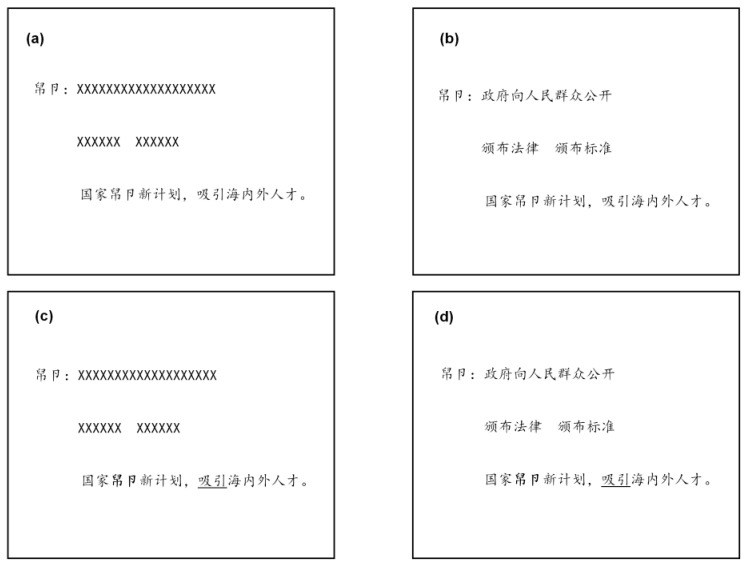
Schematic illustrations of the four instructional-support conditions used in the eye-tracking learning study. Panels (**a**–**d**) depict No Cueing, Verbal Cueing, Physical Cueing, and Full Cueing conditions, respectively. Note: “帠卪” is the target pseudoword, which replaced the original word “颁布” (promulgate); “政府向人民群众公开” is the definition of the target word, it meaning “the government disclosure to the public.”; “颁布法律” and ”颁布标准” are collocations of the target word, meaning “to officially issue a law so that it takes legal effect” and “to officially release a standard for implementation,” respectively; “国家帠卪新计划，吸引海内外人才。” is the example sentence, meaning “The country promulgate a new plan to attract talent from home and abroad.”.

The placeholder symbols in Conditions 1 and 3 were introduced to maintain visual layout consistency across conditions; they preserved the spatial arrangement of the materials but did not equate informational load, semantic richness, or reading demands. This design choice was intended to control low-level perceptual factors such as text length and screen layout, so that observed differences across conditions could be attributed to the presence or absence of cue-based instructional support rather than to mere visual differences in the stimulus display. Accordingly, the four conditions are treated as differing in instructional-support functions rather than as matched on overall information load. The material layout is illustrated in Figure 1.

#### 2.3.2. Testing Phase

The three test tasks in the present study correspond to three core dimensions of lexical representation: orthographic form, semantic meaning, and lexical usage (contextual integration) (Perfetti, 2007; Perfetti & Stafura, 2014). Upon completion of the learning phase, participants completed three tests: an Orthographic Choice Task, a Semantic Priming Decision Task, and a Sentence Acceptability Judgment Task. To ensure the appropriateness of the three tasks, both previously established task formats and independent material validation procedures were used.

**Orthographic Choice Task (OCT).** The OCT was adapted from Hong and Lai (2023) and was used to assess participants’ recognition of the orthographic forms of the newly learned pseudowords. Participants were required to select, from four orthographically similar options, the target pseudoword that had appeared during the learning phase. The task comprised 24 items, with the correct option and distractors randomly arranged in each trial. All distractors were orthographically similar to the target pseudoword, instantiating one of three patterns: (a) the first character was identical to the target while the second character was orthographically similar; (b) the first character was orthographically similar to the target while the second character was identical; or (c) both characters were orthographically similar to the target. For example, participants saw the question “Which of the following words did you just study?” followed by four options: A. 帠卪, B. 帠甲, C. 帛卪, and D. 帛甲. In this example, A was the correct answer. Option B shared the same first character with the target, whereas its second character was orthographically similar to the target’s second character. Option C had an orthographically similar first character but shared the same second character with the target. Option D contained two characters that were both orthographically similar to the corresponding characters in the target. Because the distractors were systematically constructed to vary only in orthographic similarity to the target items, the task was considered to have provided evidence for the content validity for assessing orthographic form recognition.

**Semantic Priming Decision Task (SPDT).** The SPDT was also adapted from Hong and Lai (2023) and was used to assess the semantic dimension of lexical representation. For each target word, one semantically related word and one semantically unrelated word were constructed. The related and unrelated words were matched on proficiency level, and no significant difference in stroke count was found between the two sets, *t* = 0.25, *df* = 45.97, *p* = 0.80. To validate the materials, 10 international students and 10 native speakers of Chinese who did not participate in the main experiment rated the words in terms of familiarity, comprehensibility, and semantic relatedness. The results showed no significant difference between related and unrelated words in familiarity, *t* = −0.15, *df* = 40.82, *p* = 0.88, or comprehensibility, *t* = 0.03, *df* = 45.97, *p* = 0.98, whereas the difference in semantic relatedness was significant, *t* = 61.08, *df* = 43.68, *p* < 0.001. These results indicate that the related and unrelated words differed in the intended semantic relationship while being comparable in familiarity, comprehensibility, and orthographic complexity. Thus, the task provided appropriate validity evidence for assessing semantic associations of the newly learned words. The task comprised 84 trials.

**Sentence Acceptability Judgment Task (SAJT).** The SAJT was designed to assess participants’ knowledge of the usage of the target words in sentential contexts, particularly their collocational use. This task was developed on the basis of common exercise and examination formats in L2 Chinese vocabulary instruction. For each target word, one acceptable sentence and one unacceptable sentence were constructed. Both sentences were compound sentences, and their length was controlled within 14 to 15 Chinese characters. The unacceptable sentences were created by manipulating the collocational use of the target word; that is, the source of unacceptability was restricted to the inappropriate collocation of the target word rather than to unrelated grammatical, semantic, or contextual errors.

To examine the validity of the SAJT materials, 10 international students and 10 native speakers of Chinese who did not participate in the main experiment rated the sentences for comprehensibility and judged their acceptability. The results showed that participants were able to clearly distinguish acceptable from unacceptable sentences, with a mean accuracy of 95.83% for acceptable sentences and 98.13% for unacceptable sentences. In addition, no significant difference was found between native speakers and L2 learners in acceptability judgment accuracy, *t* = −1.77, *df* = 31, *p* = 0.09, suggesting that the two groups showed comparable performance in identifying the intended acceptability contrast. The high accuracy rates for both acceptable and unacceptable sentences indicate that there was no ambiguous boundary in the acceptability judgment. Because the task required binary judgments with predetermined correct answers, the scoring procedure was objective and did not involve subjective rater interpretation.

**Figure 2:**
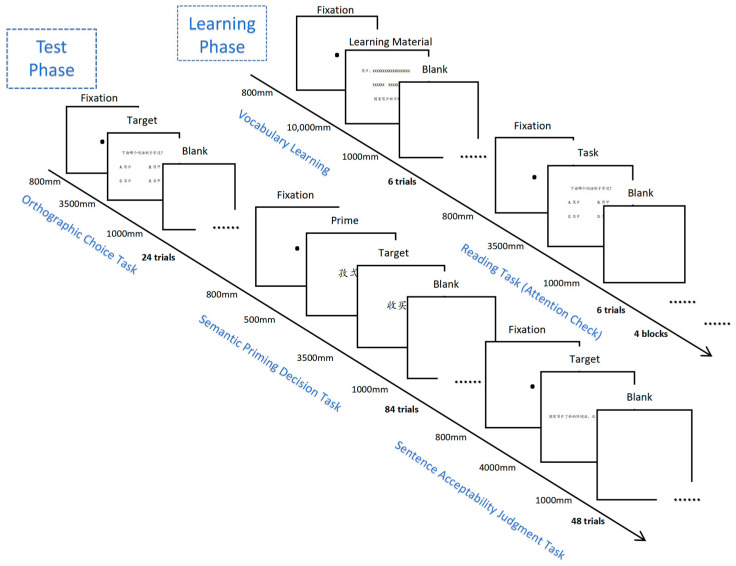
Experimental procedure of the eye-tracking study. Note: Illustration of the experimental procedure. Learning phase. Participants first studied the vocabulary learning materials, followed by a reading task serving as an attention check to ensure engagement. Test phase. Participants completed three tasks in sequence: (1) an orthographic choice task, (2) a semantic priming decision task, and (3) a sentence acceptability judgment task. The first two tasks involved lexical stimuli (Chinese characters/word pairs), whereas the third task involved sentence-level materials. The Chinese characters displayed in the figure represent sample stimuli exactly as they were presented to participants during the eye-tracking experiment. This is a schematic diagram; the Chinese text is illustrative of the actual experimental display and is not translated.

The experimental procedure is illustrated in Figure 2.

#### 2.3.3. Post-Experiment Questionnaire

To assess learners’ subjective perceptions and preferences regarding the different cueing conditions, a post-experiment questionnaire was administered upon completion of the experiment, and all participants who had completed the experiment were required to fill it in. The questionnaire comprised two sections: background information and subjective experience evaluation. The background information section collected demographic data including participants’ age, nationality, and year of study. The subjective experience evaluation section consisted of two components: first, participants rated the four types of learning materials on three dimensions—perceived difficulty, perceived helpfulness, and mental effort—using a 10-point scale; second, participants rank-ordered the four types of learning materials according to personal preference (1 = most preferred, 4 = least preferred).

### 2.4. Apparatus

Eye movements were recorded using an EyeLink 1000 eye tracker manufactured by SR Research Ltd. (Ottawa, ON, Canada), with a sampling rate of 1000 Hz. The experimental program was written in Experiment Builder. Stimuli were presented on a 19-inch computer monitor with an aspect ratio of 5:4 and a screen resolution of 1024 × 768 pixels. Target word materials were displayed in size-12 Kaiti font with triple line spacing.

### 2.5. Procedure

The experiment consisted of a learning phase and a testing phase, and was completed by each participant individually in a quiet eye-tracking laboratory. During the learning phase, a horizontal nine-point calibration procedure was employed, with a mean error of less than 0.5°. Following calibration, instructions were presented on screen, and participants completed a practice session after confirming their understanding. The formal experiment commenced once participants had become fully familiar with the procedure. The learning phase proceeded as follows: target word learning materials were presented, and the system automatically advanced to the next trial after 10 s. Each target word learning material was presented five times in a randomized order. After every six target words, participants were given an attention check question. Participants were permitted to take a rest break after every six target words and again at the end of the learning phase.

Immediately upon completion of all new word learning trials, the testing phase began. In the testing phase, participants completed four tasks: a Lexical Decision Task, an Orthographic Choice Task, a Semantic Priming Decision Task, and a Sentence Acceptability Judgment Task. A practice session preceded each task, and participants proceeded to the formal test only after becoming familiar with the response procedure.

**Figure 3:**
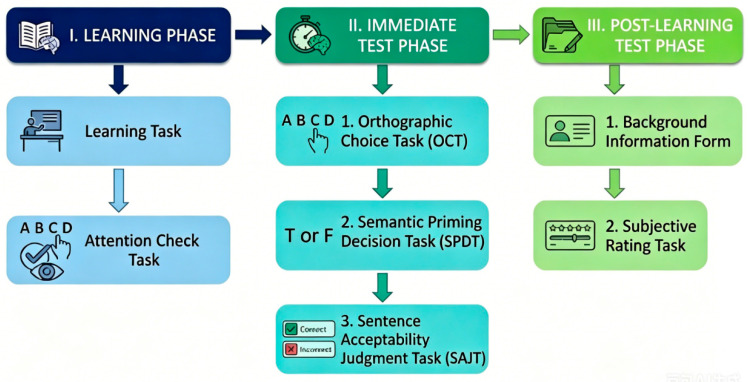
Flowchart of the experimental procedure.

In the Orthographic Choice Task, in which they were required to select the target word they had studied from four orthographically similar options. The third task was the Semantic Priming Decision Task: the target word was presented first, followed by either a semantically related or unrelated word, and participants were required to judge whether the second word was a real word, pressing “F” for yes and “J” for no. Items were presented in a randomized order, with a minimum interval of four trials between the related and unrelated words associated with the same target word. The final task was the Sentence Acceptability Judgment Task, in which participants judged whether each presented sentence was correct, pressing “F” for yes and “J” for no. A minimum interval of four trials was maintained between the acceptable and unacceptable sentences associated with the same target word. The keys were balanced between subjects. The testing phase lasted approximately 20 min. The overall experimental procedure is illustrated in Figure 3.

### 2.6. Eye-Tracking Measures

The analysis of participants’ cognitive processing during vocabulary learning comprised two components: target-word fixation measures and global learning-trial processing measures.

**(1) Target word fixation data.** With the target word in the example sentence defined as the interest area (IA), the following eye-tracking measures were selected: IA First-Fixation Duration (IA FFD), IA First-Run Dwell Time (IA FRDT), IA Dwell Time (IA DT), IA Fixation Count (IA FC), and IA Average Fixation Pupil Size (IA AFPS).

IA First-Fixation Duration refers to the duration of the first fixation made during the first pass through a given interest area. This measure is widely regarded as a key eye-tracking index reflecting early-stage lexical processing, primarily indexing the initial recognition of the target word—including sensitivity to surface-level features such as word frequency, stroke count, and orthographic complexity, as well as early processes such as orthographic recognition (Rayner, 1998; Rayner et al., 1996).

IA First-Run Dwell Time refers to the sum of durations of all fixations made within an interest area during the first pass. This measure is generally considered to reflect early-stage lexical processing and serves as an important index of the initial recognition, retrieval, and processing of the target word (Rayner, 1998; Just & Carpenter, 1980).

IA Dwell Time refers to the total duration of all fixations within the interest area, encompassing fixations made during both the initial reading pass and any regressions. This measure is typically taken to reflect relatively late-stage cognitive processing, and is particularly associated with semantic integration, resolution of processing difficulty, and reanalysis of information (Rayner, 1998; Clifton et al., 2007).

IA Fixation Count refers to the total number of fixations directed at the interest area. This measure is commonly used to reflect the cognitive load associated with processing the reading material. In general, a higher fixation count indicates that the region may entail greater processing difficulty or require greater investment of cognitive resources to achieve comprehension (Rayner, 1998; Just & Carpenter, 1980). Both IA Dwell Time and IA Fixation Count are late-stage processing indices, reflecting deeper semantic processing, integration of word and sentence meaning, and information integration (Rayner, 1998; Clifton et al., 2007; Winke et al., 2013).

IA Average Fixation Pupil Size refers to the mean pupil diameter recorded while a participant fixates within a given interest area. This measure is widely regarded as an important physiological index of cognitive effort and processing load; increases in pupil diameter are generally interpreted as indicating greater investment of cognitive resources during processing (Beatty & Kahneman, 1966; Beatty, 1982; Kahneman, 1973).

**(2) Target word learning material processing data.** Analyses at the level of the learning trial were conducted as global analyses. Trial Total Fixation Duration and Trial Total Fixation Count were selected as dependent variable indices to examine participants’ cognitive resource allocation during reading of the overall learning materials (Lai et al., 2013; Alemdag & Cagiltay, 2018).

Trial Total Fixation Duration refers to the total fixation time for a given trial within the currently selected interest period—that is, the sum of all fixation durations—and serves as an important index of reading depth, processing difficulty, and degree of cognitive engagement (Irwin, 2004; Rayner, 2009).

Trial Total Fixation Count refers to the total number of fixations recorded for a given trial within the currently selected interest period, and provides a global reflection of the total cognitive effort expended by learners in acquiring the target vocabulary item (Lai et al., 2013; Alemdag & Cagiltay, 2018).

### 2.7. Data Preprocessing

Data cleaning was performed according to the following steps. First, trials with incorrect responses were excluded from all datasets except those used for accuracy analyses. Second, for continuous variables, the first quartile (Q_1_) and third quartile (Q_3_) were computed to derive the interquartile range (IQR); data points falling below Q_1_ − 1.5 × IQR or above Q_3_ + 1.5 × IQR were identified as potential outliers and excluded. Third, eye-tracking data were additionally cleaned according to established criteria (Blythe et al., 2012; Chang et al., 2020): fixations shorter than 80 ms were removed, and eye-tracking data associated with track loss were excluded. Following these procedures, 4.22% of all trials were removed because they failed to meet the inclusion criteria.

### 2.8. Statistical Analysis

All data analyses were conducted in the R environment (R Development Core Team) using the lme4 and afex packages for model fitting. For continuous variables, log transformation was applied prior to modeling with linear mixed-effects models (LMMs). For binary and count data, generalized linear mixed-effects models (GLMMs) were constructed using logistic regression with a logit link and Poisson regression, respectively.

All linear mixed-effects models (LMMs) were fitted using the mixed() function from the afex package (Version 1.5.0). The significance of fixed effects was evaluated using Type III tests based on Satterthwaite’s approximation of denominator degrees of freedom (Luke, 2017). All generalized linear mixed-effects models (GLMMs) were fitted using the glmer() function from the lme4 package (Version 1.1.37). The significance of fixed effects was assessed using Wald z tests, which are large-sample asymptotic tests that assume infinite denominator degrees of freedom (Bates et al., 2015). Post hoc pairwise comparisons were corrected for multiple comparisons using the Tukey HSD method and implemented with the emmeans package (Version 1.11.2.8). All analyses were conducted in the R environment (Version 4.5.1).

In all models, cueing condition (with four levels: no cueing, verbal cueing, physical cueing, and full cueing) was entered as a fixed factor, while participant and item were specified as random factors. Separate models were fitted for the test outcomes and the eye-tracking outcomes, respectively. Main effects of the fixed factor were subsequently examined using Type III sums of squares. The significance threshold for all statistical tests was set at α = 0.05.

For the subjective questionnaire data, which involved a smaller number of items, rating scores were analyzed using repeated-measures analysis of variance (ANOVA); where applicable, all ANOVA analyses were corrected for violations of sphericity using the Greenhouse–Geisser method, and multiple comparisons were corrected using Tukey’s method. For the preference ranking data, which were ordinal in nature and derived from a within-subjects design, a Friedman test was used to compare preference rankings across the four conditions; upon obtaining a significant omnibus result, pairwise post hoc comparisons were conducted using the Wilcoxon signed-rank test, with Holm correction applied for multiple comparisons.

## 3. Results

The condition comparisons in the Section 3 were interpreted in relation to the information-load structure described in Section 2.2. Comparisons within the same information-load level provided relatively more controlled evidence regarding the added contribution of physical cueing, whereas comparisons across information-load levels involved differences in both the cue format and lexical–semantic information.

### 3.1. Learning Test Results

The mean accuracy on the reading comprehension questions was 97%, indicating that participants read the learning materials attentively.

**Figure 4:**
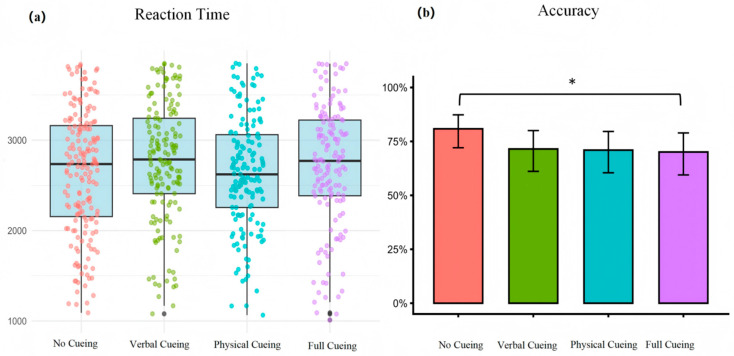
Descriptive performance for the OCT task. (**a**) Reaction time (RT, ms) distribution (boxplot with individual data points overlaid). (**b**) Accuracy (%) (bar plot with 95% confidence intervals). Black dots in boxplots represent outliers falling beyond 1.5 × IQR. This applies to all subsequent figures using the same plotting format. Note: Dots represent individual observations, and boxplots show the median and interquartile range. Significance bars indicate Tukey-adjusted post hoc pairwise comparisons. * *p* < 0.05. Only significant pairwise comparisons are shown. The same notation applies to subsequent figures unless otherwise stated.

**Figure 5:**
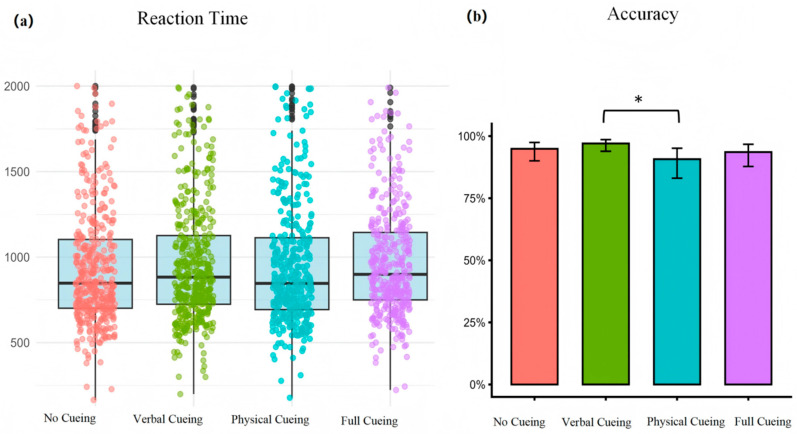
Descriptive performance for the SPDT task. (**a**) Reaction time (RT, ms) distribution (boxplot with individual data points overlaid). (**b**) Accuracy (%) (bar plot with 95% confidence intervals).Note: Dots represent individual observations, and boxplots show the median and interquartile range. Significance bars indicate Tukey-adjusted post hoc pairwise comparisons. * *p* < 0.05. Only significant pairwise comparisons are shown. The same notation applies to subsequent figures unless otherwise stated.

**Figure 6:**
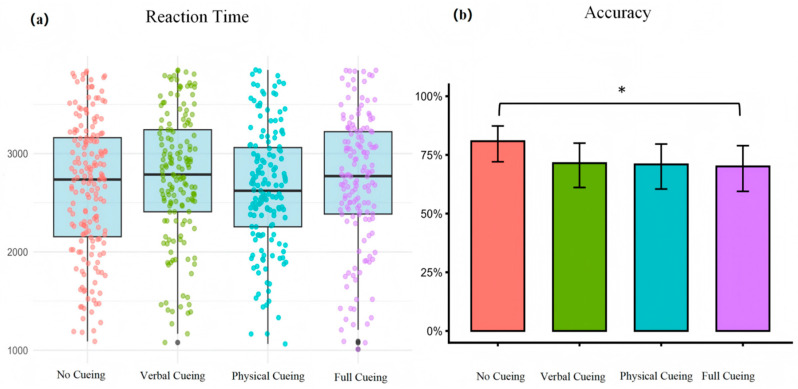
Descriptive performance for the SAJT task. (**a**) Reaction time (RT, ms) distribution (boxplot with individual data points overlaid). (**b**) Accuracy (%) (bar plot with 95% confidence intervals). Note: Dots represent individual observations, and boxplots show the median and interquartile range. Significance bars indicate Tukey-adjusted post hoc pairwise comparisons. * *p* < 0.05. Only significant pairwise comparisons are shown. The same notation applies to subsequent figures unless otherwise stated.

Based on the testing procedures described above, the reaction time and accuracy data were obtained for L2 learners’ performance on the Orthographic Choice Task, the Semantic Priming Decision Task, and the Sentence Acceptability Judgment Task under the four cueing conditions. Descriptive statistics for all three tasks across cueing conditions are presented in Table 1, and the overall descriptive performance is illustrated in Figure 4, Figure 5 and Figure 6; the inferential analysis results for reaction times are reported in Table 2, and the inferential analysis results for accuracy are reported in Table 3.

#### 3.1.1. Orthographic Choice Task Results

The results for the reaction times on the Orthographic Choice Task revealed that the main effect of the cueing condition was not significant, *F*(3, 599.95) = 1.55, *p* = 0.20. the Results for the accuracy on the Orthographic Choice Task revealed that the main effect of the cueing condition was significant, *χ*^2^(3) = 8.61, *p* = 0.04. Post hoc pairwise comparisons based on the estimated marginal means from the GLMM with a Tukey adjustment indicated that only the difference between the no cueing and full cueing conditions reached significance, *OR* = 1.80, *z* = 2.61, *p* = 0.045, with the accuracy in the no cueing condition being higher than that in the full cueing condition; all remaining pairwise comparisons were non-significant, *ps* ≥ 0.075.

#### 3.1.2. Semantic Priming Decision Task Results

The results for the reaction times on the Semantic Priming Decision Task revealed that the differences among cueing conditions did not reach significance, *F*(3, 43.56) = 0.46, *p* = 0.709. The results for the accuracy revealed that the main effect of the cueing condition was significant, *χ*^2^(3) = 8.33, *p* = 0.040. Post hoc pairwise comparisons based on the estimated marginal means from the GLMM with a Tukey adjustment indicated that the difference between the verbal cueing and physical cueing conditions was significant, *OR* = 3.36, *z* = 2.83, *p* = 0.024, with the accuracy in the verbal cueing condition being higher than that in the physical cueing condition; all remaining pairwise comparisons were non-significant, *ps* ≥ 0.237.

#### 3.1.3. Sentence Acceptability Judgment Task Results

The results for the reaction times on the Sentence Acceptability Judgment Task revealed that the main effect of the cueing condition was not significant, *F*(3, 940.89) = 0.30, *p* = 0.83. The results for the accuracy likewise revealed no significant differences among cueing conditions, *χ*^2^(3) = 0.59, *p* = 0.90.

### 3.2. Eye-Tracking Results During the Learning Phase

**Figure 7:**
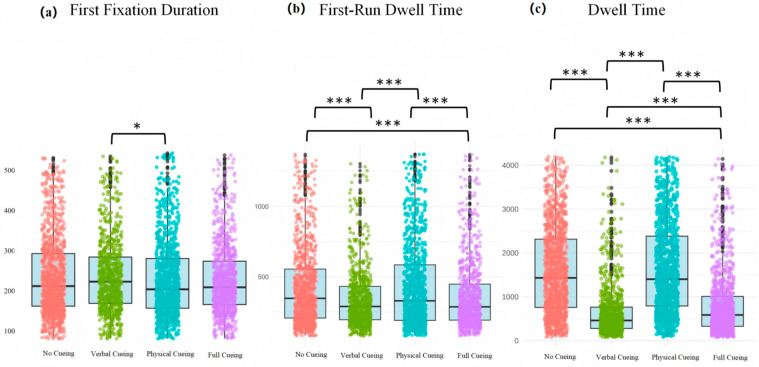
Interest-area (IA) eye-tracking measures across cueing conditions. Dots show individual observations overlaid on boxplots. Panels (**a**–**c**) depict first-fixation duration, first-run dwell time, and dwell time. Note: Dots represent individual observations, and boxplots show the median and interquartile range. Significance bars indicate Tukey-adjusted post hoc pairwise comparisons. * *p* < 0.05, *** *p* < 0.001. Only significant pairwise comparisons are shown. The same notation applies to subsequent figures unless otherwise stated.

**Figure 8:**
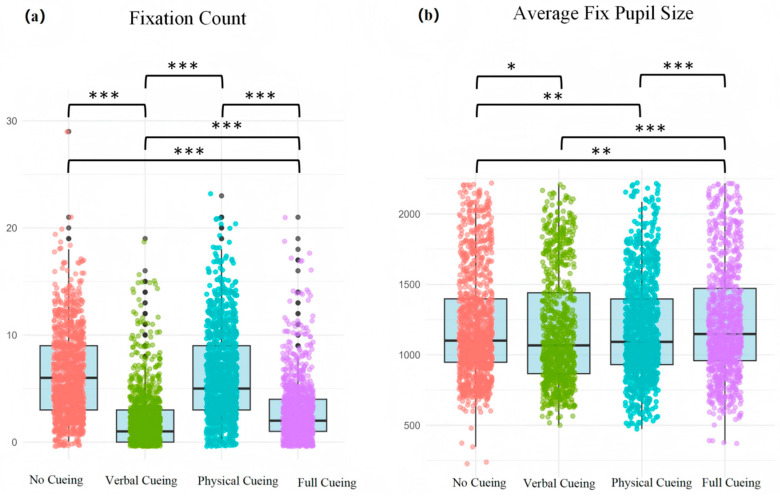
Interest-area (IA) eye-tracking measures across cueing conditions. Dots show individual observations overlaid on boxplots. Panels (**a**) depict fixation count; panels (**b**) depict average fixation pupil size. Note: Dots represent individual observations, and boxplots show the median and interquartile range. Significance bars indicate Tukey-adjusted post hoc pairwise comparisons. * *p* < 0.05, ** *p* < 0.01, *** *p* < 0.001. Only significant pairwise comparisons are shown. The same notation applies to subsequent figures unless otherwise stated.

Descriptive statistics (means) for the eye-tracking measures of the target word interest area (IA) during the learning phase are presented in Table 4, and the corresponding inferential analysis results are reported in Table 5; the distribution of each measure across cueing conditions is illustrated in Figure 7 and Figure 8.

#### 3.2.1. Target Word Fixation Data

(1) First-Fixation Duration

The main effect of the cueing condition was significant, *F*(3, 3595.36) = 3.27, *p* = 0.02. Post hoc comparisons revealed that first-fixation duration in the physical cueing condition was significantly shorter than that in the verbal cueing condition (*p* = 0.031); the difference between the physical cueing and no cueing conditions was marginally significant (*p* = 0.068), with the first-fixation duration in the no cueing condition potentially being longer than that in the physical cueing condition. All remaining pairwise comparisons were non-significant (*ps* > 0.05).

(2) First-Run Dwell Time

The main effect of the cueing condition was significant, *F*(3, 3571.11) = 12.94, *p* < 0.001. The first-run dwell time in the full cueing condition was significantly shorter than that in the no cueing condition (*p* < 0.001) and physical cueing condition (*p* < 0.001), but did not differ significantly from the verbal cueing condition (*p* = 1.000). First-run dwell times in both the no cueing and physical cueing conditions were significantly longer than that in the verbal cueing condition (*ps* < 0.001), whereas the difference between the no cueing and physical cueing conditions was non-significant (*p* = 0.87).

(3) Dwell Time

The main effect of cueing condition was significant, *F*(3, 3787.86) = 455.42, *p* < 0.001. The dwell time in the full cueing condition was significantly shorter than that in the no cueing and physical cueing conditions (*ps* < 0.001), but significantly longer than that in the verbal cueing condition (*p* < 0.001). Furthermore, dwell times in both the no cueing and physical cueing conditions were significantly longer than that in the verbal cueing condition (*ps* < 0.001), whereas the difference between the no cueing and physical cueing conditions did not reach significance, though it approached marginal significance (*p* = 0.061).

(4) Fixation Count

The main effect of the cueing condition on the fixation count was significant, *Wald χ*^2^(3) = 1916.80, *p* < 0.001. Tukey-adjusted post hoc comparisons showed that the fixation counts were significantly higher in the no cueing condition than in the verbal cueing condition, rate ratio = 3.07, *p* < 0.001, and the full cueing condition, rate ratio = 2.30, *p* < 0.001. The no cueing and physical cueing conditions did not differ significantly, rate ratio = 1.02, *p* = 0.90. Fixation counts were also significantly higher in the physical cueing condition than in the verbal cueing condition, rate ratio = 3.01, *p* < 0.001, and the full cueing condition, rate ratio = 2.26, *p* < 0.001. Finally, fixation counts were significantly higher in the full cueing condition than in the verbal cueing condition, rate ratio = 1.33, *p* < 0.001.

(5) Average Fixation Pupil Size

The main effect of cueing condition was significant, *F*(3, 3822.87) = 18.02, *p* < 0.001. The average fixation pupil size in the full cueing condition was significantly larger than that in the no cueing condition (*p* = 0.005), the verbal cueing condition (*p* < 0.001), and the physical cueing condition (*p* < 0.001). Furthermore, the average fixation pupil size in the physical cueing condition was significantly smaller than that in the no cueing condition (*p* = 0.003), and the no cueing condition showed a significantly larger average pupil size than the verbal cueing condition (*p* = 0.025), whereas the difference between the verbal cueing and physical cueing conditions was non-significant (*p* = 0.98).

#### 3.2.2. Target Word Learning Material Processing Data

**Figure 9:**
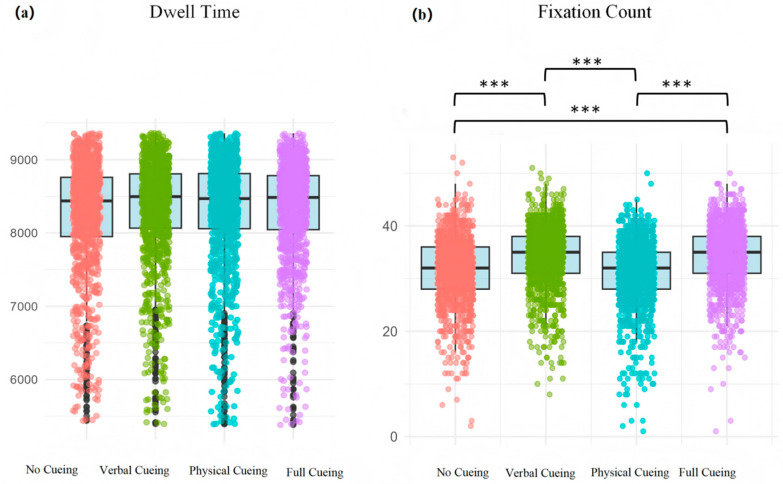
Distributions of trial-level eye-tracking measures across cueing conditions. Dots represent individual trial observations overlaid on boxplots. Panel (**a**) shows trial dwell time (ms), and panel (**b**) shows trial fixation count. Note: Dots represent individual observations, and boxplots show the median and interquartile range. Significance bars indicate Tukey-adjusted post hoc pairwise comparisons. *** *p* < 0.001. Only significant pairwise comparisons are shown. The same notation applies to subsequent figures unless otherwise stated.

The descriptive statistics for eye-tracking measures of the target word learning materials during the learning phase are presented in Table 6, and the corresponding inferential analysis results for cueing conditions are reported in Table 7; the distribution of trial-level eye-tracking measures across cueing conditions is illustrated in Figure 9.

(1) Trial Dwell Time

The main effect of cueing condition was not significant, *F*(3, 4242.86) = 2.10, *p* = 0.10.

(2) Trial Fixation Count

The main effect of the cueing condition on the trial fixation count was significant, *Wald χ*^2^(3) = 360.67, *p* < 0.001. Post hoc comparisons revealed that the fixation count in the full cueing condition was significantly higher than that in the no cueing and physical cueing conditions (*ps* < 0.001), but did not differ significantly from the verbal cueing condition (*p* = 0.28). Furthermore, fixation counts in both the no cueing and physical cueing conditions were significantly lower than that in the verbal cueing condition (*ps* < 0.001). The difference between the no cueing and physical cueing conditions did not reach significance (*p* = 0.051); however, the fixation count in the physical cueing condition was numerically slightly lower than that in the no cueing condition.

### 3.3. Post-Experiment Questionnaire Results

Based on the post-experiment questionnaire, the following section presents an analysis of the participants’ subjective experience under the four cueing conditions, encompassing their ratings of perceived learning difficulty, perceived helpfulness, and mental effort, as well as their preference rankings for the four types of learning materials.

**Figure 10:**
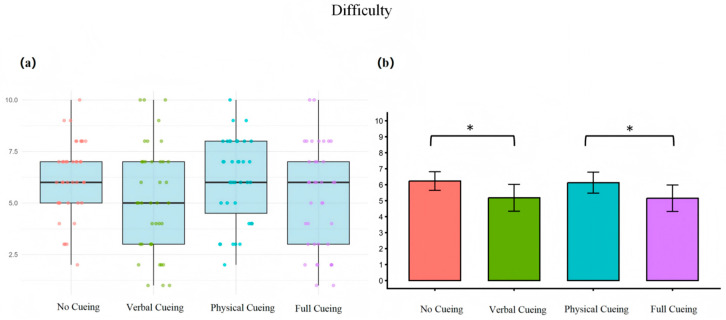
Post-experiment questionnaire ratings for difficulty across cueing conditions. (**a**) Distribution of ratings (boxplot with individual data points overlaid). (**b**) Mean ratings with 95% confidence intervals. Note: Dots represent individual observations, and boxplots show the median and interquartile range. Significance bars indicate Tukey-adjusted post hoc pairwise comparisons. * *p* < 0.05. Only significant pairwise comparisons are shown. The same notation applies to subsequent figures unless otherwise stated.

**Figure 11:**
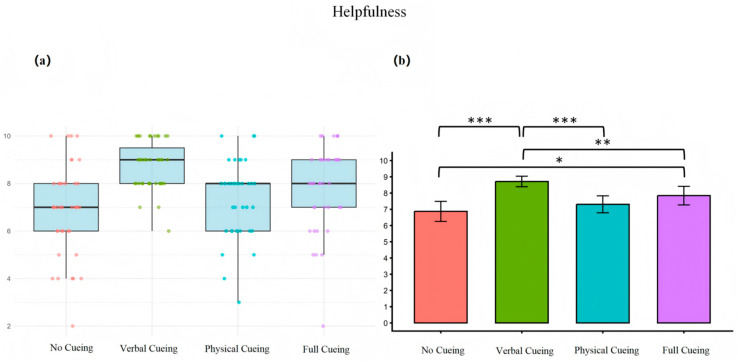
Post-experiment questionnaire ratings for helpfulness across cueing conditions. (**a**) Distribution of ratings (boxplot with individual data points overlaid). (**b**) Mean ratings with 95% confidence intervals. Note: Dots represent individual observations, and boxplots show the median and interquartile range. Significance bars indicate Tukey-adjusted post hoc pairwise comparisons. * *p* < 0.05, ** *p* < 0.01, *** *p* < 0.001. Only significant pairwise comparisons are shown. The same notation applies to subsequent figures unless otherwise stated.

**Figure 12:**
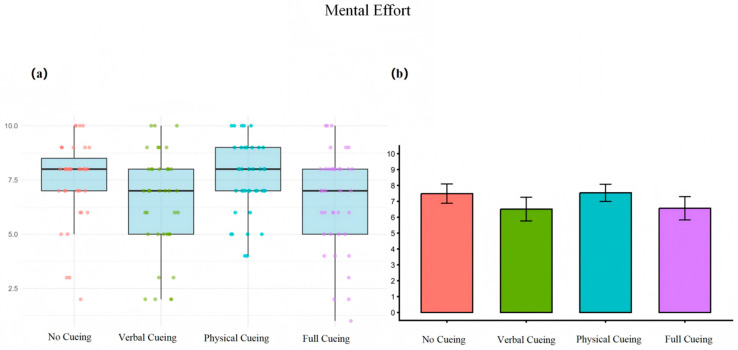
Post-experiment questionnaire ratings for mental effort across cueing conditions. (**a**) Distribution of ratings (boxplot with individual data points overlaid). (**b**) Mean ratings with 95% confidence intervals.

The descriptive statistics for the first part of the subjective experience ratings (difficulty, helpfulness, and mental effort) under the different cueing conditions are presented in Table 8; the corresponding inferential analysis results for cueing conditions are reported in Table 9; and the distributions and mean estimates are illustrated in Figure 10, Figure 11 and Figure 12.

#### 3.3.1. Difficulty

The results indicated that the main effect of the material type was significant, *F*(2.11, 80.07) = 5.71, *p* = 0.004. In terms of means, the difficulty ratings were higher in the no cueing (*M* = 6.23) and physical cueing (*M* = 6.13) conditions, and lower in the verbal cueing (*M* = 5.18) and full cueing (*M* = 5.15) conditions. Post hoc comparisons revealed that the difficulty rating in the no cueing condition was significantly higher than that in the verbal cueing condition (*p* = 0.035), and the rating in the physical cueing condition was significantly higher than that in the full cueing condition (*p* = 0.032); all remaining pairwise comparisons did not reach significance.

#### 3.3.2. Helpfulness

The results indicated that the main effect of the material type was significant, *F*(3, 95.96) = 12.39, *p* < 0.001. In terms of means, the verbal cueing condition received the highest helpfulness rating (*M* = 8.72), followed by the full cueing condition (*M* = 7.85), the physical cueing condition (*M* = 7.31), and the no cueing condition (*M* = 6.87). Post hoc comparisons revealed that the verbal cueing condition was rated significantly higher than the no cueing condition (*p* < 0.001), the physical cueing condition (*p* < 0.001), and the full cueing condition (*p* = 0.010); in addition, the full cueing condition was rated significantly higher than the no cueing condition (*p* = 0.048); all remaining pairwise comparisons were non-significant.

#### 3.3.3. Mental Effort

The results indicated that the main effect of the material type was significant, *F*(2.32, 88.14) = 5.13, *p* = 0.005. In terms of means, the mental effort ratings were lower in the verbal cueing (*M* = 6.51) and full cueing (*M* = 6.56) conditions, and higher in the no cueing (*M* = 7.49) and physical cueing (*M* = 7.54) conditions. Post hoc comparisons revealed that no pairwise comparison reached significance (*ps* ≥ 0.051). Several comparisons approached significance, including verbal cueing versus physical cueing (*p* = 0.051), physical cueing versus full cueing (*p* = 0.063), and no cueing versus verbal cueing (*p* = 0.069).

#### 3.3.4. Preference Ranking

**Figure 13:**
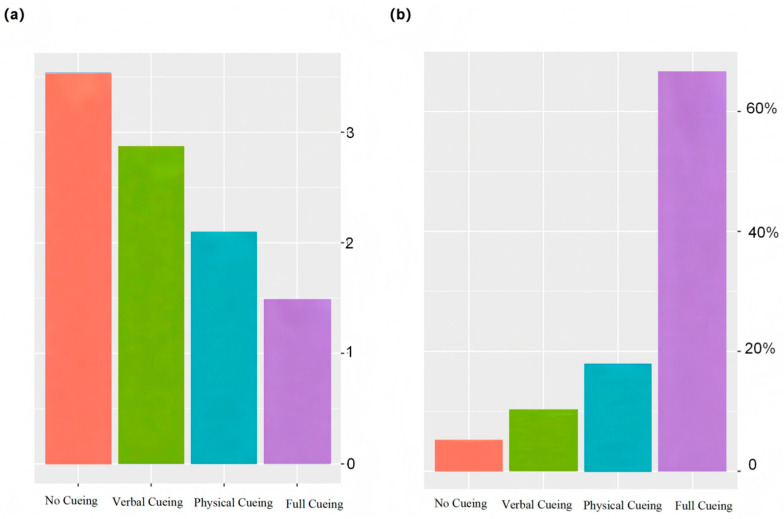
Subjective preference rankings for the four cue conditions. (**a**) Mean rank for each condition (1 = most preferred; 4 = least preferred; lower values indicate higher preference). (**b**) Proportion of participants ranking each condition as their first choice (*N* = 39).

The descriptive statistics for participants’ preference rankings of the four types of learning materials by priority in the second part of the subjective experience questionnaire are presented in Table 10, and the distribution of subjective preference rankings is illustrated in Figure 13.

An analysis of the questionnaire ranking data indicated that participants’ preferences differed significantly across the four cueing conditions. The descriptive statistics revealed the following mean rank ordering: full cueing received the lowest mean rank (*M* = 1.49), followed by verbal cueing (*M* = 2.10), physical cueing (*M* = 2.87), and no cueing (*M* = 3.54). The distribution of first-choice selections followed the same pattern: 66.67% of participants (26/39) ranked the full cueing materials as their first choice, markedly higher than any other condition.

The Friedman test indicated a significant difference in preference rankings across the four conditions, *χ*^2^(3) = 56.17, *p* < 0.001, Kendall’s W = 0.48, suggesting a medium-to-large effect for preference differences. Post hoc pairwise comparisons revealed that all pairwise differences among the four conditions reached significance (all *ps* < 0.05 after Holm correction), with the overall preference ordering consistently emerging as follows: full cueing > verbal cueing > physical cueing > no cueing (where “>“ denotes greater preference).

## 4. Discussion

The present study examined how different forms of cue-based instructional support—no cueing, verbal cueing, physical cueing, and full cueing—were associated with L2 Chinese learners’ immediate vocabulary outcomes, online visual processing, and subjective evaluations. Rather than showing a uniform facilitative effect, the results revealed differentiated patterns across orthographic form, semantic meaning, and sentence-level usage judgment. Importantly, these effects should be interpreted in light of both the cueing format and information-load differences across conditions.

### 4.1. Do Instructional-Support Conditions Modulate Chinese L2 Vocabulary Processing and Immediate Outcomes?

The overall findings indicate that cue-based instructional support affected learners’ immediate vocabulary outcomes and online processing in a measure-dependent manner. Instead, the observed patterns varied across lexical dimensions, task types, and information-load levels. Consequently, the results should be interpreted in relation to both the cue format and the amount of lexical–semantic information provided, rather than as reflecting pure cueing effects.

With respect to orthographic form retention, cueing conditions influenced learning outcomes but did not produce a stable facilitative effect. On the contrary, the no cueing condition yielded the highest accuracy, while the full cueing condition yielded the lowest. This suggests that the addition of cues does not necessarily benefit the retention of orthographic information; rather, the introduction of supplementary information may divert learners’ attention away from the target word form itself.

With respect to semantic comprehension, the verbal cueing condition outperformed the physical cueing condition, indicating that cues capable of directly supplying meaning and collocation information are more conducive to helping learners establish form–meaning mappings. This finding demonstrates that, compared to physical cueing, verbal cueing exerts a more pronounced facilitative effect on semantic comprehension, representing one of the clearer manifestations of cueing efficacy observed in the present study.

With respect to lexical usage, no significant differences emerged among cueing conditions, suggesting that, under cue-supported learning, learners had not yet developed stable differences in their mastery of lexical usage. This indicates that, under the short-term repeated exposure conditions of the present study, cues offered relatively a limited facilitation of higher-order vocabulary knowledge.

Eye-tracking data further showed that several indices of online processing were sensitive to the instructional-support condition. Multiple eye-tracking measures were significantly influenced by the cueing condition, indicating that different cueing configurations did alter learners’ attentional allocation and processing strategies. In other words, the effects of cueing were reflected not only in the final test performance, but also in the adjustment of processing pathways during learning.

Subjective evaluations from the post-experiment questionnaire also supported the existence of cueing effects. Compared to the no cueing and physical cueing conditions, learners overall expressed a greater preference for verbal cueing and full cueing materials, and rated these two types of materials as more helpful, easier to understand, and requiring less mental effort. In other words, cueing influenced not only objective learning outcomes but also learners’ subjective experience of the materials. However, this subjective preference did not reliably correspond to advantages in objective learning outcomes or online processing efficiency. This divergence suggests that learners’ subjective perception of helpfulness or ease did not necessarily correspond to reduced online processing demands, especially in the full cueing condition.

Taken together, the cueing effects observed in the present study are better characterized as a modulation of learners’ information processing, rather than a straightforward improvement in performance. This finding is consistent with the core tenets of the Cognitive Theory of Multimedia Learning and the signaling principle, which hold that cues facilitate the selection, organization, and integration of information by directing learners’ attention toward key content (Mautone & Mayer, 2001; de Koning et al., 2009). Such modulation does not occur under conditions of unlimited cognitive resources, but is constrained by learners’ finite cognitive capacity. When cues enhance the directionality of attention toward critical information, they may facilitate effective resource allocation; however, when cues themselves introduce additional processing demands, they may also consume limited cognitive resources and thereby attenuate learning (Kahneman, 1973; Navon & Gopher, 1979; Sweller, 2024). This resource competition and coordination demand may partly explain the relatively small effect sizes (e.g., *η_p_*^2^ < 0.01) observed in certain behavioral and eye-tracking measures in the present study. At the same time, the present study did not find that cues consistently improved learning performance across all tasks, which is not fully consistent with studies emphasizing the universal facilitative effects of cueing on the learning outcomes. The present findings are instead more in line with the research that has highlighted the context-dependence of cueing effects, noting their susceptibility to task characteristics, material complexity, and cognitive resource constraints (Kriz & Hegarty, 2007; Boucheix & Lowe, 2010; de Koning et al., 2007; Schneider et al., 2018). It is therefore evident that, while cueing effects were manifested at the overall level, their influence was not uniformly distributed across all learning outcomes; rather, they exhibited differential patterns as a function of the dimension of vocabulary knowledge under consideration. Building on this, the following sections discuss in greater detail the specific roles of different cue types with respect to orthographic form retention, semantic comprehension, and lexical usage.

### 4.2. Differential Effects of Cue Types Across Dimensions of Vocabulary Learning

The findings provided partial support for H1 and H2. Verbal cueing showed a clearer advantage in semantic comprehension, whereas physical cueing mainly facilitated early visual orienting but did not lead to stable improvements in orthographic performance. The three test tasks in the present study correspond to three core dimensions of lexical representation: orthographic form, semantic meaning, and lexical usage (contextual integration), as operationalized through measures of orthographic form retention, semantic comprehension, and lexical application, respectively (Perfetti, 2007; Perfetti & Stafura, 2014). Having established the overall presence of cueing effects, the present study further reveals that the influence patterns of different cue types were not uniform across these three dimensions.

With respect to orthographic form retention, the results did not indicate a stable facilitative advantage for physical cueing. Behavioral results showed that the no cueing condition yielded the highest accuracy on the Orthographic Choice Task, while the full cueing condition yielded the lowest, suggesting that the addition of cues does not necessarily confer an advantage when the primary learning objective involves orthographic retention. The eye-tracking results revealed that the first-fixation duration on the target word was the shortest in the physical cueing condition, indicating that salience-enhancing cues such as bolding and underlining were capable of attracting attention more rapidly and facilitating target word recognition during early visual processing. However, this early orienting advantage did not translate into higher offline orthographic performance, suggesting that the facilitative effects of physical cueing on orthographic learning may be confined primarily to the perceptual level—enabling rapid localization—without reliably strengthening subsequent orthographic representation. This finding indicates that the advantage of physical cueing is largely manifested in early visual orienting and does not necessarily extend to the formation of high-quality orthographic representations.

By contrast, the verbal cueing and full cueing conditions involved greater amounts of supplementary information, which may have required learners to redistribute their limited cognitive resources between form processing and meaning processing, thereby affecting the retention of orthographic information to some extent (Barcroft, 2002; Chandler & Sweller, 1991; Harp & Mayer, 1998). This interpretation is broadly consistent with the findings of the present study: on the one hand, the no cueing condition yielded the highest accuracy on the Orthographic Choice Task while the full cueing condition yielded the lowest; on the other hand, the verbal cueing and full cueing conditions were associated with more fixations on the overall learning materials, yet a lower total dwell time and fixation count on the target word interest area. This suggests that their processing pattern was more likely characterized by frequent switching and integration between the target word and the supplementary information, rather than sustained attention on the target word itself. Such a processing mode—involving switching and integration among the target word, example sentence, and additional cue information—may be more conducive to meaning integration, but not necessarily to the fine-grained encoding of orthographic information. Consequently, in learning tasks where orthographic form is the primary objective, reducing supplementary information may not diminish learning effectiveness; on the contrary, it may be more beneficial for maintaining focused attention on the word form itself.

With respect to semantic comprehension, the results suggest a relative advantage of linguistic–semantic support, although this pattern should be interpreted in light of the information-load structure of the design. When information load was held relatively constant—that is, when Condition 2 (verbal cueing/linguistic–semantic cueing) was compared with Condition 4 (full cueing/combined cueing), both of which presented definitions and collocations—semantic judgment accuracy in the verbal cueing condition did not differ significantly from that in the full cueing condition (*M* = 0.94 vs. *M* = 0.88). This comparison indicates that adding physical/perceptual cueing to existing linguistic–semantic support did not provide clear additional benefits for immediate semantic performance.

By contrast, verbal cueing produced significantly higher semantic judgment accuracy than physical cueing (*M* = 0.94 vs. *M* = 0.84). However, this comparison crosses information-load levels, because the verbal cueing condition provided definitions and collocations whereas the physical cueing condition did not. Therefore, this difference should not be interpreted as a pure cueing effect. Rather, it suggests that, under the present instructional conditions, learners benefited from the lexical–semantic information supplied by definitions and collocations.

Taken together, these findings indicate that linguistic–semantic support was more closely aligned with the demands of semantic comprehension than perceptual cueing alone. Compared with physical cueing, which primarily enhanced the perceptual salience of the target word, definitions and collocations provided information more directly relevant to meaning construction and form–meaning mapping. This interpretation is consistent with recent glossing research showing that the richness and contextualization of gloss information can shape vocabulary learning outcomes. For example, Rassaei and Folse (2024) found that sentence-level L2 glosses produced stronger vocabulary learning gains than word-level L1 or L2 glosses, suggesting that contextualized semantic support may enable learners to construct more elaborated lexical representations. It is also consistent with the recent meta-analytic evidence showing that textual and multimedia glosses can support L2 vocabulary learning, especially when the outcome measure taps recognition or meaning-related performance (Zhang & Ma, 2024; Mahdi et al., 2024).

In semantic comprehension tasks, what is critical is not only whether learners notice the target word, but whether they can use effective informational support to establish connections among word form, conceptual meaning, semantic features, and related collocations. Recent studies on glossing and collocational learning similarly suggest that collocation-level support may shape lexical and collocational learning differently from word-level or purely perceptual enhancement (Jung et al., 2024). Thus, the observed advantage should be interpreted as the effect of linguistic–semantic instructional support rather than as verbal cueing functioning as a pure signaling mechanism. Physical cueing in the present study may indeed have enhanced early attention to the target word; yet, this attentional advantage did not automatically translate into a higher semantic accuracy. This suggests that, in semantic learning, if a cue fails to provide meaning-relevant support, its function may remain confined to the level of perceptual orienting, without advancing deeper semantic construction. This interpretation is also consistent with recent evidence that typographic enhancement can increase perceptual salience and support some forms of collocational learning, but that its effects depend on the type of target construction and may not generalize uniformly across lexical outcomes (Toomer et al., 2024).

The eye-tracking results were broadly consistent with this interpretation. The total dwell time and total fixation count on the target word interest area were significantly lower in the verbal cueing condition than in all other conditions; the full cueing condition, while significantly lower than the no cueing and physical cueing conditions, remained significantly higher than the verbal cueing condition. In conjunction with the higher semantic judgment accuracy observed under verbal cueing, this suggests that learners may have required the less repeated inspection of the target word when explicit semantic and collocational information was available. The higher dwell time and fixation count in the full cueing condition relative to the verbal cueing condition may further suggest that adding perceptual cues to linguistic–semantic support introduced additional coordination demands. However, the reduced dwell time and fewer fixations should not be taken as direct evidence of more efficient learning, because they may also reflect differences in the processing depth or the strategic redistribution of attention to other parts of the learning material. This ambiguity in eye-tracking interpretation aligns with the broader finding of the present study: the relationship between online processing patterns and offline learning outcomes is not straightforward and depends on multiple factors, including task demands, cue configuration, and informational load. This result aligns with the view that cueing effects are task-dependent—that is, the effectiveness of a cue is largely determined by the degree to which the information it provides is relevant to the current learning objective (Schneider et al., 2018).

From the perspective of L2 vocabulary learning, this finding also suggests that semantic learning is not a mechanical memorization of individual word forms, but rather a process of progressively building semantic connections and lexical relationships. Definitional information can help learners more rapidly integrate new words into their existing conceptual systems, while collocational information further delimits the typical semantic contexts and usage tendencies of the target word, enabling the semantic representation to develop beyond a vague and isolated form toward a more precise and retrievable one (Boers & Lindstromberg, 2009; Nation, 2022). The advantage of the verbal cueing condition in the present study can therefore be understood as arising from the combined provision of definitions and collocations, which, together, offered relatively comprehensive semantic support—enabling learners not only to grasp the conceptual meaning of the target word, but also to more readily understand its typical semantic scope and its associative relationships with other words. At the same time, the present findings are not fully consistent with those of certain prior studies suggesting that enhancing the salience of target vocabulary items can promote attentional allocation and, under some conditions, improve the associated learning performance (Choi, 2017; Jung et al., 2025a; Schneider et al., 2018). This suggests that, in semantic learning, if a cue fails to provide meaning-relevant support, its function may remain confined to the level of perceptual orienting, without advancing deeper semantic construction.

With respect to lexical usage, the differences among cue types did not emerge in immediate behavioral outcomes. In the Sentence Acceptability Judgment Task, no significant differences were found among the four cueing conditions on either reaction time or accuracy, indicating that learners’ mastery of lexical usage under cue-supported learning had not yet manifested a stable differentiation. Compared to orthographic recognition and semantic retrieval, lexical usage involves higher-order vocabulary knowledge, encompassing not only the mastery of conceptual meaning but also the integration of collocational relationships, grammatical constraints, contextual appropriateness, and conditions of use (Richards, 1976; Nation, 2022). Accordingly, the acquisition of usage-level knowledge typically proceeds more slowly than that of form and meaning, and generally requires more extensive contextual processing, greater numbers of encounters, and repeated integration (Henriksen, 1999; Nation, 2022; Webb, 2007).

From the perspective of lexical representation quality, high-quality lexical representations require not only that learners acquire word form and meaning, but also that stable, rapidly retrievable, and contextually applicable connections are formed among these elements (Perfetti & Stafura, 2014). In the present study, while the verbal cueing and full cueing conditions may have facilitated immediate comprehension, this facilitation was more reflective of support for meaning processing and information integration, and was not sufficient to further translate into stable lexical usage competence after a brief learning session. In other words, a developmental gap remains between learners’ understanding of word meaning and their ability to accurately judge lexical usage—a gap that requires continued processing and consolidation. Prior research has similarly demonstrated that different dimensions of vocabulary knowledge develop at different rates, and that collocational and usage knowledge in particular tends to develop more slowly (Webb, 2007). Consequently, the effects of cueing on lexical usage learning may only become detectable over longer learning periods, with richer contextual input, or through more sensitive productive measures.

Taken together, the different cue types exerted differential effects across dimensions of vocabulary learning. Compared to physical cueing, verbal cueing demonstrated a more unambiguous advantage on the semantic dimension. Although the full cueing condition outperformed the no cueing and physical cueing conditions on certain online processing indices, it did not exhibit a stable additional advantage over the verbal cueing condition. On the orthographic dimension, the addition of cues did not produce a stable facilitation and may even have weakened the focused attention on word form through informational competition. As for lexical usage, the current results have not yet revealed significant differences. These findings suggest that cueing effects do not constitute a uniform, homogeneous facilitative phenomenon; rather, they exhibit differential patterns as a function of learning objectives and levels of lexical representation. More specifically, physical cueing appears to operate primarily at the information selection stage, where it more readily confers advantages in early visual orienting; verbal cueing is more likely to support information organization and integration, and, therefore, exerts a more pronounced facilitative effect on the semantic dimension; and higher-order lexical usage may require longer learning periods, richer contextual input, and greater numbers of repeated exposures before the stable benefits of cueing become apparent.

It is further worth noting that the present study not only reveals differential effects of cue types across dimensions, but also demonstrates that the concurrent presentation of multiple cues did not produce a simple additive outcome. This warrants a further discussion of why the additional effects in the full cueing condition were unstable, and why the combination of multiple cues failed to yield linearly cumulative benefits.

### 4.3. Limited Additive Benefits of the Full Cueing Condition and Increased Coordination Demands

Based on the research hypotheses, the full cueing condition was expected to outperform single-cue conditions; however, the results provided only partial support for H3.

Most critically, the comparison between Condition 2 (verbal cueing/linguistic–semantic cueing) and Condition 4 (full cueing/combined cueing) provides the most direct test of whether adding physical/perceptual cueing to existing linguistic–semantic support yields additional benefits, because these two conditions contained the same definitions and collocations. The results did not provide evidence for such additive benefits. On semantic judgment accuracy, the verbal cueing condition did not differ significantly from the full cueing condition (*M* = 0.94 vs. *M* = 0.88). On orthographic accuracy, the full cueing condition also did not outperform the verbal cueing condition and was numerically lower (*M* = 0.66 vs. *M* = 0.70). Thus, when the amount of lexical–semantic information was comparable across conditions, adding physical/perceptual cueing did not improve immediate vocabulary performance. This finding suggests that more cue-based support does not necessarily lead to better learning outcomes under the present experimental conditions.

The full cueing condition performed relatively well on subjective helpfulness ratings and material preference rankings, but did not demonstrate a stable additional advantage in objective learning outcomes, indicating that the effects of cueing are not simply additive.

More specifically, the full cueing condition did not significantly outperform the verbal cueing condition on semantic judgment accuracy; on the orthographic task, the full cueing condition, in fact, performed the most poorly; during online processing, the first-fixation duration in the full cueing condition was not superior to that in the physical cueing condition, and the pupil size indices were the highest, indicating greater processing costs. At the same time, although the total dwell time and total fixation count on the target word interest area were lower in the full cueing condition than in the no cueing and physical cueing conditions, they remained significantly higher than in the verbal cueing condition. It is therefore evident that, under combined cueing conditions, the learning outcomes did not accumulate linearly with the addition of cues; rather, the additional benefits of full cueing over verbal cueing appeared limited.

This is not inconsistent with the broader trends reported in the cueing literature. Meta-analytic findings have demonstrated that cues generally promote retention and transfer and, under certain conditions, reduce the cognitive load; however, these effects are not stable and are modulated by factors such as the nature of the learning materials, the form of the cues, the outcome measures employed, and the demands of the task (Schneider et al., 2018; Xie et al., 2017). Furthermore, the present findings are not fully consistent with studies that have reported immediate advantages for combinatorial enhancement. For example, F. Wang et al. (2020) found that both visual cues and text-visual combined cues facilitated transfer performance and the organization and integration of information. By contrast, the full cueing condition in the present study did not consistently outperform the verbal cueing condition, suggesting that the simultaneous presence of multiple cues does not automatically translate into stronger learning outcomes. The effectiveness of combined cueing depends on whether the individual cues form genuine complementarity, rather than merely accumulating in terms of presentational form.

From a theoretical standpoint, this result is more consistent with a coordination-demand or resource-competition account than with a simple additive-cueing account. Because physical cueing and verbal cueing did not provide fully overlapping information, the present findings should not be interpreted as direct evidence of a redundancy effect in the strict CLT sense. Cognitive Load Theory and redundancy effect research have indicated that, when learners have already acquired the core information necessary for comprehension, the additional presentation of information that overlaps with the learning objective—or that is supplementary but of limited value—may instead consume finite working memory resources, increase extraneous processing demands, and thereby attenuate learning outcomes (Chandler & Sweller, 1991; Kalyuga et al., 1999). In the present study, verbal cueing had already provided learners with the two types of core support most directly relevant to meaning construction: definitions and collocations. The further addition of physical cues such as bolding and underlining may not have supplied new effective support; rather, it is likely to have increased the coordination demands placed on learners as they navigated among the target word, example sentence, definition, collocation, and typographic markers. Under these circumstances, the physical cues in the full cueing condition may no longer function as helpful prompts, but instead become additional processing objects of limited incremental value.

This interpretation also helps explain why the full cueing condition yielded an inconsistent performance across dimensions. For semantic learning, verbal cueing was already sufficiently capable of supporting form–meaning mapping and integration, leaving little additional contribution for the superimposed physical cues. For orthographic learning, the full cueing condition required learners to simultaneously process form, meaning, and additional typographic markings; the resulting resource competition readily manifested as a detrimental effect on the fine-grained encoding of orthographic information, which accounts for the paradoxical finding of the highest informational load being associated with the lowest orthographic accuracy. At the same time, the larger pupil size indices observed in the full cueing condition suggest that a greater resource investment does not necessarily yield higher-quality learning: if those resources were devoted primarily to coordinating among supplementary information elements rather than directly to the construction of target word representations, the learning returns would be expected to be limited (Xie et al., 2017).

The present study therefore converges with the perspectives of the Cognitive Theory of Multimedia Learning and Cognitive Load Theory: the effectiveness of cueing depends not on the quantity of cues per se, but on the degree to which they are aligned with the learning objective, the unique informational value they provide, and the manner in which learners allocate their limited cognitive resources. Only when an additional cue provides irreplaceable support can a multi-cue combination be expected to yield genuine additive effects; otherwise, the returns are likely to be limited and may increase coordination demands rather than improve learning. It should be noted, however, that the present study’s examination of cueing effects and their boundaries remains preliminary, and there remains considerable scope for further development in terms of research design, measurement approaches, and task contexts.

### 4.4. Limitations and Future Directions

Although the present study systematically compared the effects of no cueing, physical cueing, verbal cueing, and full cueing on intentional Chinese L2 vocabulary learning, and examined processing differences across cueing conditions through the integration of eye-tracking data, behavioral outcomes, and subjective evaluations, several methodological limitations warrant discussion. Some of these limitations reflect the inherent trade-offs in experimental design, while others suggest avenues for future refinement.

First, an important methodological limitation concerns the information-load structure of the four cueing conditions. As explained in Section 1.3 and Section 2.2, the four conditions differed not only in the cueing format but also in the amount and nature of lexical–semantic information provided. Specifically, the no cueing and physical cueing conditions were minimally informative, whereas the verbal cueing and full cueing conditions provided definitions and collocational information. This structure allowed relatively clearer comparisons within the same information-load level, such as the comparison between the no cueing and physical cueing conditions, and the comparison between the verbal cueing and full cueing conditions. However, comparisons across information-load levels necessarily confound the cueing format with informational content. Therefore, the differences in eye movements and immediate learning outcomes across these conditions may partly reflect the differences in semantic richness, reading demands, or content availability, rather than cueing mechanisms alone. Accordingly, the effects observed in the verbal cueing and full cueing conditions should be interpreted as effects of linguistic–semantic instructional support rather than as pure cueing effects.

Second, another limitation concerns participant heterogeneity in first-language background. Although all participants were learners from non-character-based language backgrounds, they came from multiple L1 backgrounds. Differences in L1 background may be associated with the variation in Chinese collocational processing, vocabulary learning, or responsiveness to different forms of cueing and instructional support. This possibility is consistent with recent evidence that the character- and word-level features in Chinese may pose different levels of difficulty for learners from different L1 backgrounds (Yan & Lin, 2023). Due to the limited sample size, the present study was not able to conduct meaningful subgroup analyses or examine L1-related variation. Future research with larger and more balanced samples should compare learners from different L1 backgrounds to clarify whether and how L1 background moderates the effects of cue-based instructional support.

Third, the present study relied primarily on immediate post-tests. Consequently, the findings are more reflective of learners’ initial processing, short-term lexical retention, and representational states following brief learning sessions, rather than durable vocabulary acquisition. The absence of delayed post-tests makes it difficult to determine whether the observed effects of different cueing conditions would persist over time or support long-term vocabulary retention and delayed retrieval.

Fourth, although vocabulary learning outcomes were examined across three dimensions—orthographic form, semantic meaning, and lexical usage—the assessment battery was predominantly composed of receptive tasks. As a result, the learners’ productive lexical knowledge remains insufficiently examined. Future studies should therefore incorporate productive measures, such as sentence construction, contextualized gap-filling, oral production, or written composition, to provide a more sensitive assessment of the productive and usage-related dimensions of lexical knowledge.

Fifth, the operationalization of cueing in the present study was limited to verbal cueing, physical cueing, and their combination. Other cueing features discussed in the multimedia learning literature, such as cue intensity, density, position, timing, complexity, color highlighting, spatial contiguity, and gestural cues, were beyond the scope of the present investigation. Therefore, the boundary conditions governing the effectiveness of different cueing designs remain insufficiently specified. In addition, the operational definitions used in the present study may differ from those employed in other cueing research, which may limit the direct comparability with prior studies.

Sixth, the present study employed a tightly controlled experimental task context that differs in certain respects from vocabulary learning as it occurs in authentic classroom instruction and naturalistic reading. The use of controlled materials and fixed exposure conditions helped reduce potential confounds, but it may also limit the ecological validity of the findings. In natural learning contexts, learners often encounter vocabulary through repeated exposure, self-paced reading, teacher explanation, peer interaction, and communicative use. Therefore, the extent to which the present findings generalize to authentic instructional settings requires further examination.

In light of these limitations, future research may extend the present work in several directions. First, future studies should further disentangle the cueing format from the information load by adopting more fully orthogonal experimental designs in which perceptual cueing, linguistic–semantic information, and textual load are independently manipulated or carefully matched. For example, future studies could independently manipulate the presence of linguistic–semantic information and typographical enhancement while matching the textual length, visual complexity, and reading demands across conditions. Such designs would help determine whether learning gains are driven primarily by perceptual cueing, linguistic–semantic information, their combination, or the interaction between cueing format and information load. Second, future studies should also recruit larger and more balanced samples from different L1 backgrounds, which would make it possible to examine whether learners’ first-language background moderates Chinese collocational processing, vocabulary learning outcomes, or the effectiveness of different cueing conditions. Third, the inclusion of delayed post-tests would enable an examination of the effects of different cueing conditions on vocabulary retention and long-term acquisition, thereby providing a more comprehensive account of the durability of cueing effects. Fourth, the addition of productive tasks such as sentence construction and contextualized gap-filling would allow for the more sensitive detection of the facilitative effects of cueing on the pragmatic dimension of lexical knowledge. Fifth, future research could systematically manipulate cue intensity, density, position, timing, and complexity, thereby providing clearer quantitative boundaries and design principles for multimedia vocabulary instruction. Finally, examining different cueing designs in classroom-based instruction, self-paced digital reading, multimedia vocabulary learning, or naturalistic reading environments would enhance the ecological validity and pedagogical applicability of the findings.

In summary, the present study represents an important initial step in comparing different forms of cue-based instructional support in L2 Chinese vocabulary learning. However, several design and methodological features limit the scope and generalizability of the findings. Most notably, the confounding of the cueing format with the information load requires a cautious interpretation of the comparisons across information-load levels, while within-load comparisons should be understood as relatively more interpretable rather than fully isolated tests of cueing mechanisms. Future research that addresses these limitations would further clarify the principles governing effective cueing in multimedia vocabulary instruction.

## 5. Conclusions

The present study compared four instructional-support conditions—no cueing, physical cueing, verbal cueing, and full cueing—to examine how different forms of cue-based support were associated with learners’ immediate L2 Chinese vocabulary learning, online visual processing, and subjective evaluations. The findings suggest that cue-based instructional support did not exert a uniform facilitative effect. Instead, its effects varied according to the type of support provided, the dimension of vocabulary knowledge being assessed, and the processing demands imposed by the learning materials.

The null findings for the Sentence Acceptability Judgment Task (SAJT) should be interpreted with caution. As an offline measure, the SAJT likely draws on relatively stable and explicit collocational knowledge, whereas the knowledge acquired during a brief learning session may remain emergent and insufficiently consolidated. This mismatch may explain why early collocational gains were not captured by the SAJT.

Importantly, these findings should be considered in light of the functional differences among the cueing conditions, which varied in both cueing format and information load. The physical cueing in the present study is primarily of typographical enhancement and can thus be regarded as a form of perceptual cueing carrying minimal lexical–semantic information. By contrast, verbal cueing supplied definitions and collocational information and can therefore be characterized as linguistic–semantic instructional support than as a purely perceptual cueing mechanism. From this perspective, the relatively clearer advantage of verbal cueing on semantic comprehension may reflect not only attentional guidance, but also the additional lexical–semantic information it made available. Its benefits, then, are best understood as effects of lexical–semantic support rather than as evidence for a pure cueing effect.

The results further suggest that adding more cues did not necessarily produce stronger immediate learning. Although the full cueing condition was more favorably received in terms of subjective helpfulness and material preference, it showed no stable advantage over the verbal cueing condition on objective learning outcomes or online processing indices. This pattern indicates that the effectiveness of cue-based support depends not simply on the number of cues, but on the extent to which different forms of support provide distinct informational value and can be coordinated within learners’ limited cognitive resources. Because the conditions differed in information load as well as cueing format, however, comparisons across information-load levels warrant caution: differences between minimally informative and information-rich conditions may partly reflect the variation in semantic richness, content availability, or reading demands rather than cueing format alone.

These findings are broadly consistent with the Cognitive Theory of Multimedia Learning and Cognitive Load Theory, which hold that instructional support is most beneficial when it directs learners’ attention to task-relevant information without imposing unnecessary processing demands. The present results, however, do not support a simple “more-cues-are-better” account. Rather, they indicate that effective cueing design should consider the alignment among the cue format, informational content, learning objective, and learner processing capacity. In pedagogical terms, information-rich support such as definitions and collocations may be especially useful when the goal is to promote initial semantic comprehension. Perceptual cueing may help direct attention to target forms but may be insufficient on its own to support deeper semantic or usage-related learning. Combined cueing may benefit learners who can integrate multiple information sources; yet, it may also increase coordination demands when the added cue does not provide sufficiently distinct support.

Overall, the present study represents an initial empirical step toward understanding how perceptual cueing, lexical–semantic support, and their combination influence immediate L2 Chinese vocabulary learning. The findings, however, should be taken as evidence about the short-term learning performance and online processing under controlled experimental conditions rather than as direct evidence of durable vocabulary acquisition. Future research using factorial designs, delayed post-tests, and more ecologically valid learning contexts will be needed in order to disentangle the cueing format from the information load and to clarify the boundary conditions under which different forms of cue-based support facilitate L2 vocabulary development.

## Figures and Tables

**Table 1:** Vocabulary test scores in the learning test across conditions.

Condition	Orthographic Choice Task		Semantic Priming Decision Task		Sentence Acceptability Judgment Task	
	RT (ms)	ACC	RT (ms)	ACC	RT (ms)	ACC
No Cueing	2659 (714)	0.78 (0.42)	926 (325)	0.90 (0.30)	5020 (1431)	0.53 (0.50)
Verbal Cueing	2784 (695)	0.70 (0.46)	950 (331)	0.94 (0.25)	5005 (1444)	0.55 (0.50)
Physical Cueing	2665 (639)	0.68 (0.47)	926 (327)	0.84 (0.37)	5081 (1392)	0.54 (0.50)
Full Cueing	2725 (704)	0.66 (0.47)	958 (304)	0.88 (0.32)	5043 (1493)	0.54 (0.50)

Note. Values are means, with standard deviations (*SD*) in parentheses; the same format applies hereafter.

**Table 2:** The effect of cueing on reaction time across tasks (OCT, SPDT, and SAJT).

Variable	Test	Predictor	*NumDF*	*DenDF*	*F*	*p*	*η_p_* ^2^
RT	Orthographic Choice Task	Cueing	3	599.95	1.55	0.20	0.008
	Semantic Priming Decision Task	Cueing	3	43.56	0.46	0.71	0.03
	Sentence Acceptability Judgment Task	Cueing	3	940.89	0.30	0.83	0.01

**Table 3:** Main effects of cueing condition on accuracy based on GLMMs.

Variable	Test	Predictor	*df*	*Wald χ* ^2^	*p*
ACC	Orthographic Choice Task	Cueing	3	8.61	0.04
	Semantic Priming Decision Task	Cueing	3	8.33	0.04
	Sentence Acceptability Judgment Task	Cueing	3	0.59	0.90

**Table 4:** Descriptive statistics for target-word interest-area (IA) eye-tracking measures across cueing conditions.

Condition	First Fixation Duration (ms)	First Run Dwell Time (ms)	Dwell Time (ms)	Fixation Count	Average Fixation Pupil Size (mm)
No Cueing	234 (97.2)	388 (230.7)	1599 (1013.8)	6.0 (3.9)	1184 (350.1)
Verbal Cueing	235 (89.6)	337 (195.9)	661 (621.9)	2.0 (2.6)	1163 (384.5)
Physical Cueing	224 (91.8)	392 (250.3)	1632 (1041.3)	6.0 (4.1)	1160 (331.3)
Full Cueing	226 (83.6)	344 (210.4)	813 (728.0)	3.0 (2.8)	1201 (362.0)

**Table 5:** Fixed effects from linear mixed-effects models for target-word interest-area (IA) eye-tracking measures.

Variable	Predictor	*df*	Test Statistic	*F*	*p*	*ηp* ^2^
First-Fixation Duration	Cueing	3	*F* = 3595.36	3.27	0.02	<0.01
First-Run Dwell Time	Cueing	3	*F* = 3571.11	12.94	<0.001	0.01
Dwell Time	Cueing	3	*F* = 3787.86	455.42	<0.001	0.27
Fixation Count	Cueing	3	*Wald χ*^2^ = 1916.80	-	<0.001	-
Average Fix Pupil Size	Cueing	3	*F* = 3822.87	18.02	<0.001	0.01

**Table 6:** Eye-tracking measures for the target-word learning materials.

Condition	Dwell Time (ms)	Fixation Count
No Cueing	8418 (580)	31.5 (6.42)
Verbal Cueing	8441 (564)	34.1 (6.12)
Physical Cueing	8464 (579)	30.9 (6.45)
Full Cueing	8411 (554)	34.5 (6.03)

**Table 7:** The fixed effect of cueing on eye-tracking measures.

Variable	Model	Predictor	Test Statistic	*DenDF*	*p*	Effect Size
Dwell Time	LMM	Cueing	*F* = 2.10	34,242.86	0.10	*η_p_*^2^ < 0.01
Fixation Count	Poisson GLMM	Cueing	*Wald χ*^2^ = 360.67	3	<0.001	IRRs reported in pairwise comparisons

**Table 8:** Descriptive statistics for post-experiment questionnaire ratings across cueing conditions (means).

Condition	Difficulty	Helpfulness	Mental Effort
No Cueing	6.23 (1.8)	6.87 (1.91)	7.49 (1.88)
Verbal Cueing	5.18 (2.59)	8.72 (1)	6.51 (2.3)
Physical Cueing	6.13 (2.03)	7.31 (1.61)	7.54 (1.67)
Full Cueing	5.15 (2.56)	7.85 (1.77)	6.56 (2.26)

**Table 9:** The main effect of cueing condition on post-experiment questionnaire ratings (difficulty, helpfulness, and mental effort) from repeated-measures ANOVA.

Variable	Predictor	*NumDF*	*DenDF*	*F*	*p*	*ηp* ^2^
Difficulty	material	2.11	80.07	5.71	0.004	0.13
Helpfulness	material	2.53	95.96	12.39	<0.001	0.25
Mental effort	material	2.32	88.14	5.13	0.005	0.12

**Table 10:** Descriptive statistics for ranking (lower mean rank indicates higher preference).

Condition	Mean Rank	*SD* of Rank
Full Cueing	1.49	0.79
Verbal Cueing	2.10	0.82
Physical Cueing	2.87	0.66
No Cueing	3.54	0.97

## Data Availability

The raw data supporting the conclusions of this article will be made available by the authors upon request.

## Appendix A

### Appendix A.1. Chinese Proficiency Test (Cloze Test)

Reproduced from Feng et al. (2020).

### Appendix A.2. Summary of the Model Random-Effects Structure Simplification Process

**Model**	**Dependent** **Variable**	**Fixed** **Effect**	**Subject** **Random Effects**	**Item** **Random Effects**	**Model Description**
model.01	log()	type	(1 + type | Name)	(1 + type | word_num)	Maximal model, including random intercepts for subjects and items, as well as random slopes for type.
model.02	log()	type	(1 + type || Name)	(1 + type || word_num)	Removed the correlation parameters between random intercepts and random slopes.
model.03	log()	type	(1 + type || Name)	(1 | word_num)	Retained the subject-level random slope for type and removed the item-level random slope for type.
model.04	log()	type	(1 | Name)	(1 | word_num)	Retained only the random intercepts for subjects and items.
